# Proteomic profiling identifies specific histone species associated with leukemic and cancer cells

**DOI:** 10.1186/s12014-015-9095-4

**Published:** 2015-08-27

**Authors:** Rajbir Singh, Sean W. Harshman, Amy S. Ruppert, Amir Mortazavi, David M. Lucas, Jennifer M. Thomas-Ahner, Steven K. Clinton, John C. Byrd, Michael A. Freitas, Mark R. Parthun

**Affiliations:** Department of Biological Chemistry and Pharmacology, The Ohio State University, Columbus, OH 43210 USA; Department of Internal Medicine, The Ohio State University, Columbus, OH 43210 USA; Department of Molecular Virology, Immunology and Medical Genetics, The Ohio State University, Columbus, OH 43210 USA; Division of Medicinal Chemistry in the College of Pharmacy, The Ohio State University, Columbus, OH 43210 USA

**Keywords:** Histone, Post-translational modification, Chromatin, Chronic lymphocytic leukemia

## Abstract

**Background:**

Chromatin is an extraordinarily complex structure. Much of this complexity results from the presence of numerous histone post-translational modifications and histone variants. Alterations in the patterns of histone post-translational modifications are emerging as a feature of many types of cancer and have been shown to have prognostic value.

**Results:**

We have applied a liquid chromatography/mass spectrometry-based approach to comprehensively characterize the histone proteome in primary samples from chronic lymphocytic leukemia (CLL) patients, as well as bladder and breast cancer cell culture models. When compared to non-malignant CD19+ B cells from healthy donors, the CLL histone proteome showed a distinct signature of differentially expressed species, spanning all the histones studied and including both post-translationally modified species and unmodified, non-allelic replication-dependent histone isoforms. However, the large changes in histone H3 and H4 that are characteristic of many cancer types were not observed. One of species of H2A (mass = 14,063 Da) was the most strongly associated with time to treatment in CLL patients. CLL patient samples also demonstrated histone profiles that were distinct from those of the bladder and breast cancer cells.

**Conclusions:**

Signatures of histone profiles are complex and can distinguish between healthy individuals and CLL patients and may provide prognostic markers. In addition, histone profiles may define tissue specific malignancies.

**Electronic supplementary material:**

The online version of this article (doi:10.1186/s12014-015-9095-4) contains supplementary material, which is available to authorized users.

## Background

The packaging of eukaryotic genomes with histones to form chromatin is essential for the necessary condensation and protection of DNA. Given the central role of histones in chromatin, there have been a number of studies that have examined histone proteins to identify alterations, particularly post-translational modifications, associated with cancer. Indeed, several of the post-translational modifications may have clinical utility as prognostic markers. For example, decreased levels of histone H4 lysine 16 acetylation (H4 K16Ac) and H4 lysine 20 trimethylation (H4 K20me3) are found in a number of human cancers, while low global levels of histone H3 lysine 9 dimethylation (H3 K9me2) and H3 lysine 18 acetylation (H3 K18Ac) predict poorer outcome in prostate, lung, kidney and pancreatic cancer [1–9].

The intricate mechanisms of regulation that involve chromatin are largely based on the enormous complexity built into this structure. While histone post-translational modifications are clearly an important source of complexity, variations in the primary sequences of specific histone proteins are also a prime contributor. There are two basic groups of histone proteins, the replication-dependent histones and the replication-independent histone variants. The replication-dependent histones are encoded by multi-gene families that are found in several distinct clusters in the human genome. The expression of these histone genes is tightly coupled to DNA replication and they serve to package DNA into chromatin during DNA replication. The replication-independent histone variants, such as histone H3.3 and histone H2AZ, are typically expressed constitutively throughout the cell cycle and are found as single genes dispersed throughout the genome. Histone variants can play an important role in carcinogenesis, where driver mutations in histone H3.3 have been identified in pediatric glioblastoma [10–12].

While primary sequence diversity in histones is often thought to be the result of the incorporation of histone variants, the multiple replication-dependent histone genes do not all encode identical proteins [13, 14]. For example, there are 16 genes that code for replication-dependent histone H2A and they produce 11 distinct polypeptides. These histone species will be referred to as histone isoforms to distinguish them from the more familiar histone variants. The replication-dependent histone isoforms typically vary from each other by a small number of amino acids. This high degree of identity makes them challenging to study using typical techniques that would require the generation of isoform-specific antibodies. However, they can be readily resolved by LC/MS due to the change in mass (see Additional file 1: Tables S1–S4). The clinical importance of histone isoforms is highlighted by recent reports that identified alterations in specific histone H2A isoforms in B cells isolated from patients with chronic lymphocytic leukemia (CLL) and in estrogen receptor positive breast cancer tissues [15–17].

We have used LC/MS to quantify the relative abundance of every detectable histone species in a large set of primary tumor samples. By comparing the histone profiles between normal B cells and B cells isolated form CLL patients, we have identified a number of specific histone species with levels significantly altered in the CLL cells. In addition, histone profiles were correlated with clinical data from CLL patients to identify species that may warrant further study. The most promising was a specific histone H2A species (14,063 MW) where expression was indicative of a shortened time to treatment. We have also comprehensively analyzed histone profiles from cell culture models of bladder and breast cancer to determine whether histone alterations are specific to CLL or whether they are a more general feature of cancer cells. Interestingly, our analyses indicated that significantly decreased levels of histone H4 lysine 16 acetylation are not a ubiquitous property of cancer cells. Our studies highlight the enormous heterogeneity of histone isoforms present in human cells and indicate the importance of quantifying the histone proteome to understand the impact of chromatin structure on carcinogenesis.

## Results

Histones were isolated from CLL patient samples (n = 87) or CD19+ cells from healthy volunteers (n = 5) by acid extraction. The extracted histones were analyzed by LC–MS as described in the methods section. For each core histone peak eluting from the LC, we selected a specific mass range that encompassed all of its isoforms in an unmodified state as well as the possible spectrum of modifications that could occur. We then tested for differential expression by determining the relative abundance of each molecular species in that mass range between CLL patient samples and normal donors as well as among varying states of disease aggression in bladder and breast cell lines. An overview of our methodology has been shown in Additional file 2: Figure S1.

### Global changes in histone H2A species in leukemic and cancer cells

#### Chronic lymphocytic leukemia

**Fig. 1:**
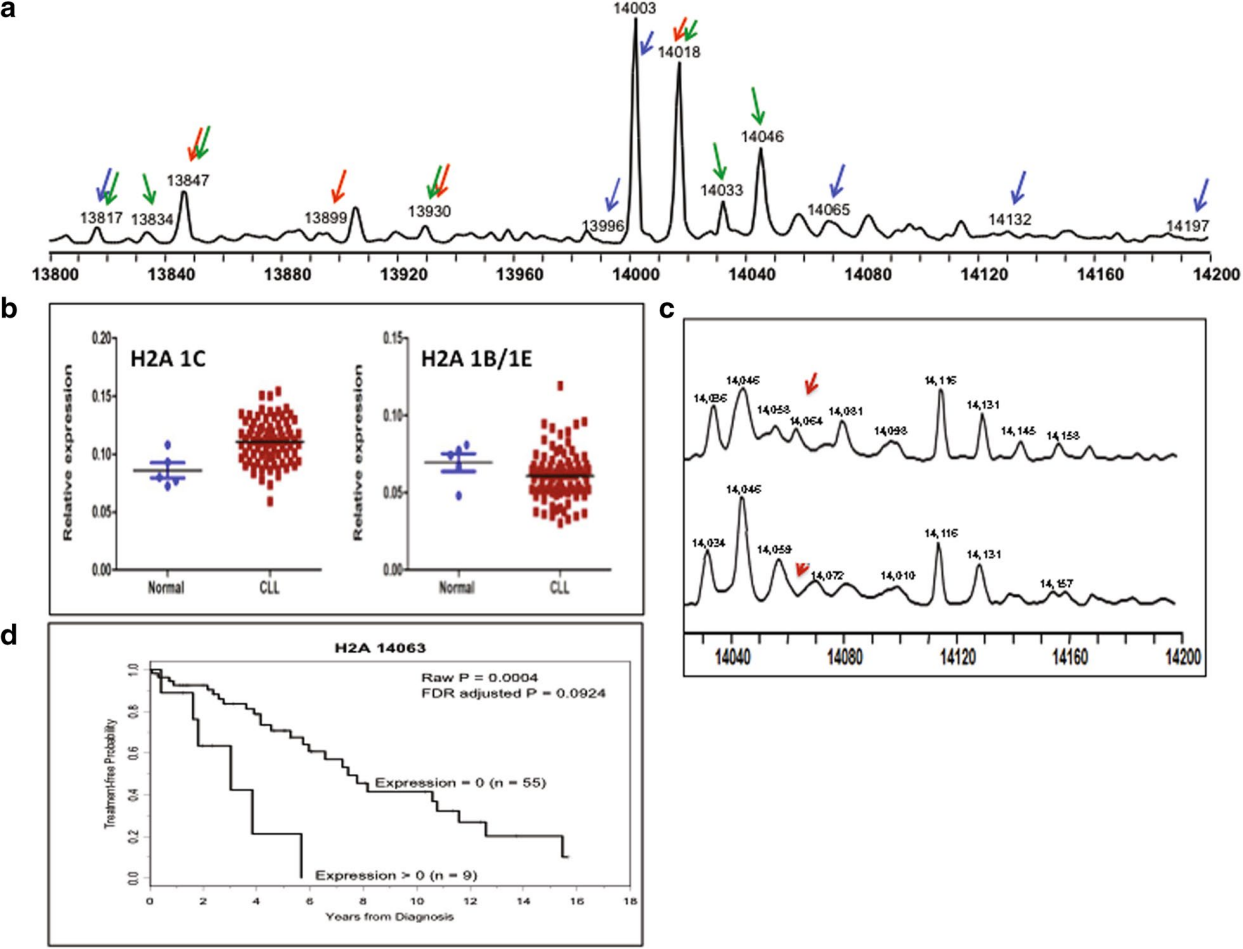
Histone H2A isoforms altered in CLL. **a** A representative spectrum histone H2A. The peaks that are significantly changed in CLL, bladder and breast cancer cells are highlighted with arrows (CLL-*green*, bladder-*red* and breast-*blue*). **b** Comparison of H2A 1C and H2A 1B/1E in normal B cells vs CLL patient B cells. **c** Representative spectrum showing the presence or absence of the peak with molecular weight 14,063 Da in healthy (*top*) CLL patient (*bottom*) B cells. **d** Kaplan–Meier curve showing the comparison of time to treatment in patients with and without the 14,063 Da peak

The unmodified replication-dependent histone H2A isoforms have masses that range from 13,817 to 14,102 (Additional file 1: Table S1). A deconvoluted spectrum of H2A from a representative normal CD19+ B cell sample is shown in Fig. 1a. Each of the peaks in the spectrum represents a histone isoform or a post-translationally modified histone isoform with a defined molecular weight. In our previous study (n = 35), we focused on the three most abundant forms of H2A, which have molecular masses of 14,002, 14,018 and 14,046 Da, and normalized each peak to the total amount of histone present in these three isoforms [15]. Given the complicated nomenclature that has arisen for the replication-dependent histones, we will use a systematic nomenclature based on the gene name for each isoform. The replication-dependent histone genes are named based on their identity and their location in the genome. The first part of the name refers to the histone cluster (e.g. HIST1, HIST2 or HIST3), the second part indicates the type of histone (e.g. H2A, H2B, H3, H4 or H1), and finally, the multiple copies of each histone type are designated alphabetically based on their order within each cluster (centromere distal to proximal). Hence, HIST1H2AC refers to the third histone H2A gene in histone cluster 1. We will refer to the protein isoforms based on this genomic nomenclature. For example, the product of the HIST1H2AC gene will be referred to as H2A 1C (for HIST**1**H2A**C**) (see Additional file 1: Tables S1–S4 for complete list of genes/protein names). Using this nomenclature, we refer to these forms as H2A, H2A 1C and H2A 1B/E, respectively. In our previous evaluation, we noted that H2A 1C and H2A 1B/E were decreased in CLL compared to non-malignant B cells [15]. In our present study, we used a larger pool of patient samples and comprehensively measured the amount of histone present across the entire region, normalizing each peak relative to the total amount of histone. Using this approach, we found that a decrease in the relative abundance of H2A 1B/E was consistently observed, however H2A 1C was present at an elevated abundance, on average, compared to the normal samples (Fig. 1b). The level of H2A 1B/E was highly variable and we observed both increased and decreased levels of this isoform in the CLL patient samples relative to the healthy controls.

Examination of all detectable H2A isoforms identified 12 that showed statistically significant changes in abundance in the CLL patient samples (Table 1), many by more than 2-fold. Based on the molecular masses of these peaks, it is not possible to definitively determine the identity of these species but they are highly modified, containing methylation and acetylation, as well as the possibility of phosphorylation. In addition, there were modest increases in the unmodified forms of canonical H2A and H2A 1H (Table 1).

**Table 1:**
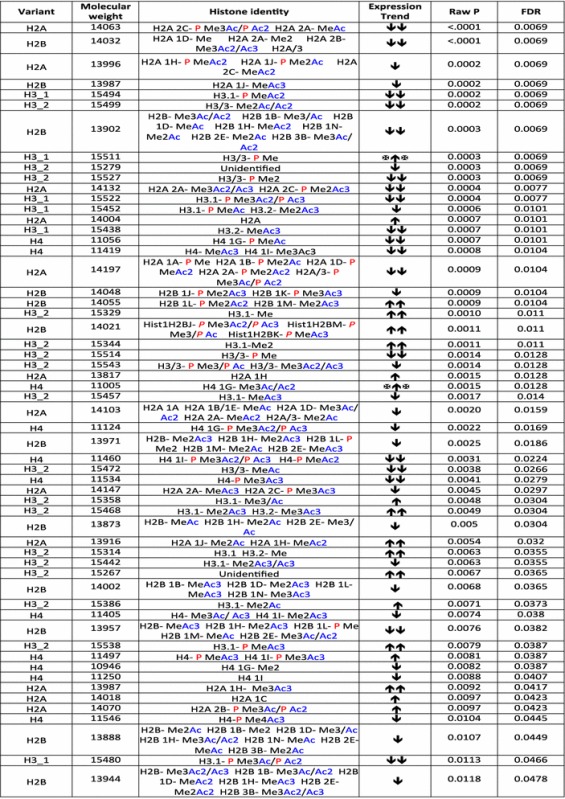
Histone isoforms that are modified in CLL samples relative to Normal samples with FDR < 0.05

The molecular weight listed may be attributed to either of the histone isoforms listed or to a mixture of these. Two-fold increases and decreases are indicated with ↑↑ or ↓↓, respectively. Increases and decreases that were less than two-fold are indicated with ↑ or ↓, respectively. Increases and decreases where the median expression for one of the groups was 0, thus prohibiting calculation of fold-changes, is indicated by  or , respectively

The most significantly altered isoform of H2A had a molecular mass of 14,063 Da (Fig. 1c). This mass is compatible with post-translationally modified versions of several H2A isoforms. For example, this mass is consistent with the H2A 1H Me3Ac3Phos1, H2A 2C Me3Ac1Phos1 or H2A 2A Me1Ac1. This species was easily detectable in all of the healthy control samples (Fig. 1c), but the CLL patient samples appeared to represent two distinct groups: one with the majority of patients who had completely undetectable levels and a second, small group with detectable levels. Intriguingly, presence of this species was associated with shorter time to treatment and was the only species with FDR adjusted p-value <0.10 (Fig. 1d). It is unclear why higher expression that is closer to normal levels would be deleterious and needs further study.

#### Bladder and breast cancer models

To determine which changes in histone profiles observed in CLL patients are disease- or tissue-specific and which might be more generally indicative of a cancerous state, we characterized the complement of histone proteins in two solid tumor model systems. The first is a set of cell lines that represent a range of histopathologic subtypes of bladder cancer. The cells that we have examined are normal bladder epithelium, normal bladder epithelial cells that have been immortalized with hTERT, a cell line derived from a non-invasive bladder cancer (RT4) and cell lines derived from invasive bladder cancers (T24 and UMUC3) [18]. Even though none of the histone species met our criteria for statistical significance, we did observe that levels of H2A 1C dropped when the normal bladder epithelial cells became immortalized with hTERT, consistent with the recent report that decreased levels of H2A 1C expression resulted in increased proliferation of cells in vitro [17].

**Fig. 2:**
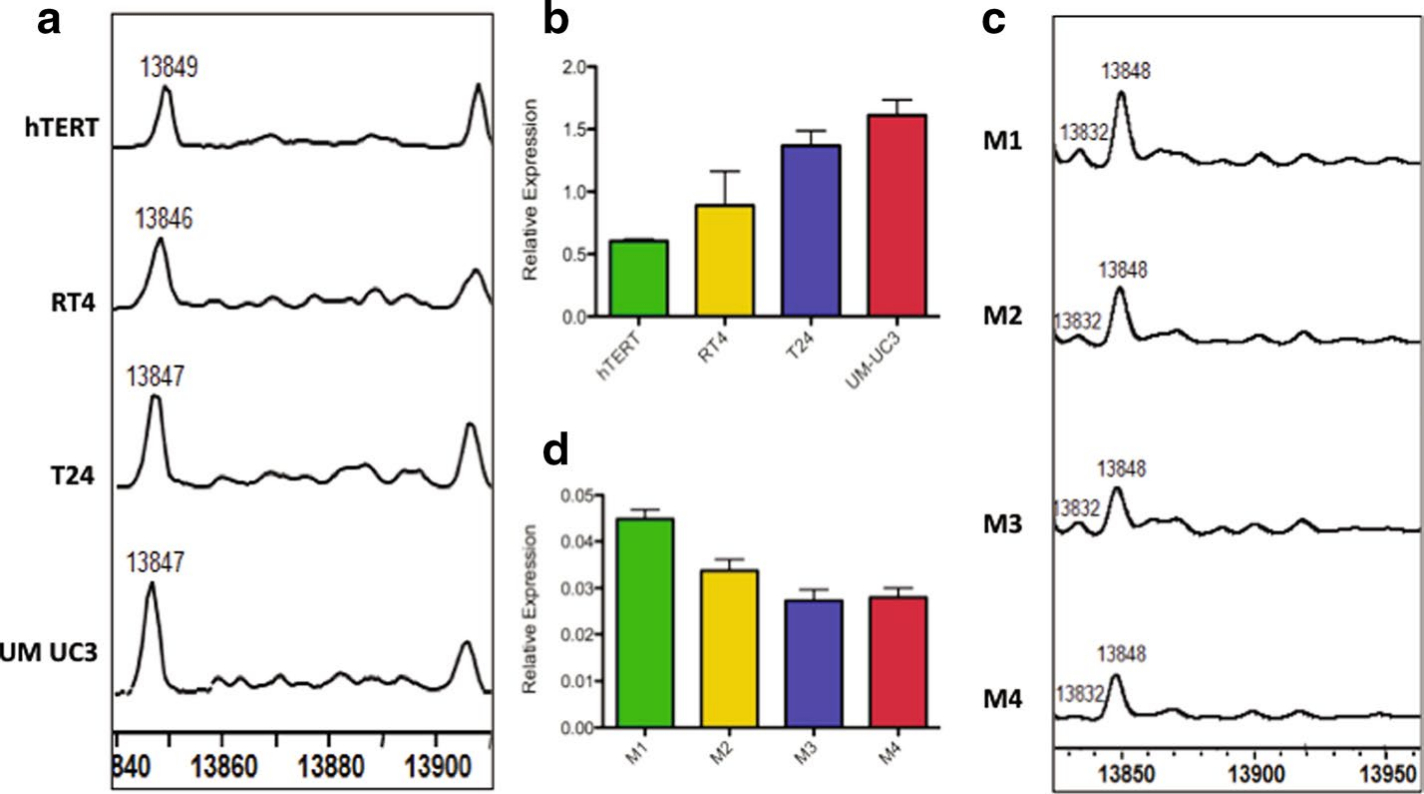
Histone H2A isoforms altered in bladder and breast cancer cells. **a** Representative spectrum of H2A 1J (molecular weight ~13,847 Da) isolated from hTERT immortalized bladder epithelium (hTERT) or the bladder cancer cell lines RT2, T24 and UMUC3 (as indicated). **b** Relative abundance of H2A 1J in the indicated bladder cell lines. Relative abundance is the ratio of the area under curve representing H2A 1J relative to the sum of the areas of all the H2A peaks. **c** Representative spectrum of H2A 1J isolated from the indicated breast cancer cell lines. **d** Plot of the relative abundance of H2A 1J in the indicated breast cancer cell lines determined as described above

Two other, less abundant, H2A species also showed decreased expression levels as the bladder cells became more invasive, including unmodified H2A 2C and an acetylated/methylated form of H2A 1J. In addition, one species, unmodified H2A 1J, displayed increased levels in the more malignant cells (Fig. 2a, b). The presence of H2A species with a molecular mass of 14,063 Da that was indicative of a poor prognosis in CLL, did not show significant changes in the bladder cancer cells suggesting that changes in this species may be tissue-specific (Additional file 1: Table S6a, b).

We also analyzed the histone complement in a set of breast cancer cell lines, designated M1, M2, M3 and M4, that represent a spectrum of subtypes found with this disease, with M1 representing immortalized normal breast epithelium, M2 representing premalignant cells, M3 representing malignant cells and M4 representing highly aggressive metastatic disease [19, 20]. The H2A spectrum is highly variable among these cell lines with 9 isoforms changing significantly (Table 2). Interestingly, the levels of H2A 1C and H2A 1B/E displayed a statistically significant increase as the cells became more tumorigenic. This observation is consistent with the recent report that increased levels of H2A 1C are seen in estrogen receptor positive breast cancers [16]. Changes in less abundant H2A species were also observed, such as the mono-methylated form of H2A 1H which decreased in abundance as the breast cancer cells became more malignant (Table 2). Notably, the unmodified form of H2A 1J, which displayed an increase in abundance in bladder cancer cell lines, showed an inverse trend in breast cancer cells (Fig. 2c, d). Similarly, the levels of H2A 1H show an inverse trend in both breast cancer cells and CLL patients (Tables 1, 2). Given the heterogeneity in different cancers and cancer cell lines, mechanistic studies are needed in order to precisely identify how these isoforms are integrated into the cancer pathway.

**Table 2:**
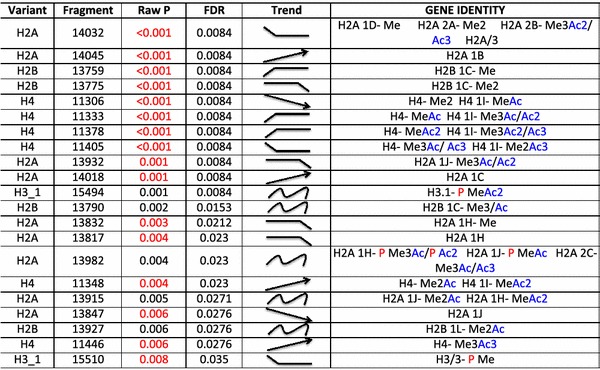
Histone isoforms that are modified among the breast cancer cell lines with FDR < 0.05

The molecular weight as listed may be attributed to either of the histone informs listed or to a mixture of these.  represents that the isoform is increased or decreased in a linear manner to cancer agression.  indicates that the isoforms are increased or decreased in more or less aggressive cell lines.  indicates that the isoform is changed in a non-linear manner among the cell lines.  represents that the level of isoform is high/low in the normal/immortalized cell line but decreases/increases in the aggressive cell lines respectively.  indicates that the expression of the isoform drops in the most aggressive cell line

### Global changes in histone H2B species in leukemic and cancer cells

#### Chronic lymphocytic leukemia

**Fig. 3:**
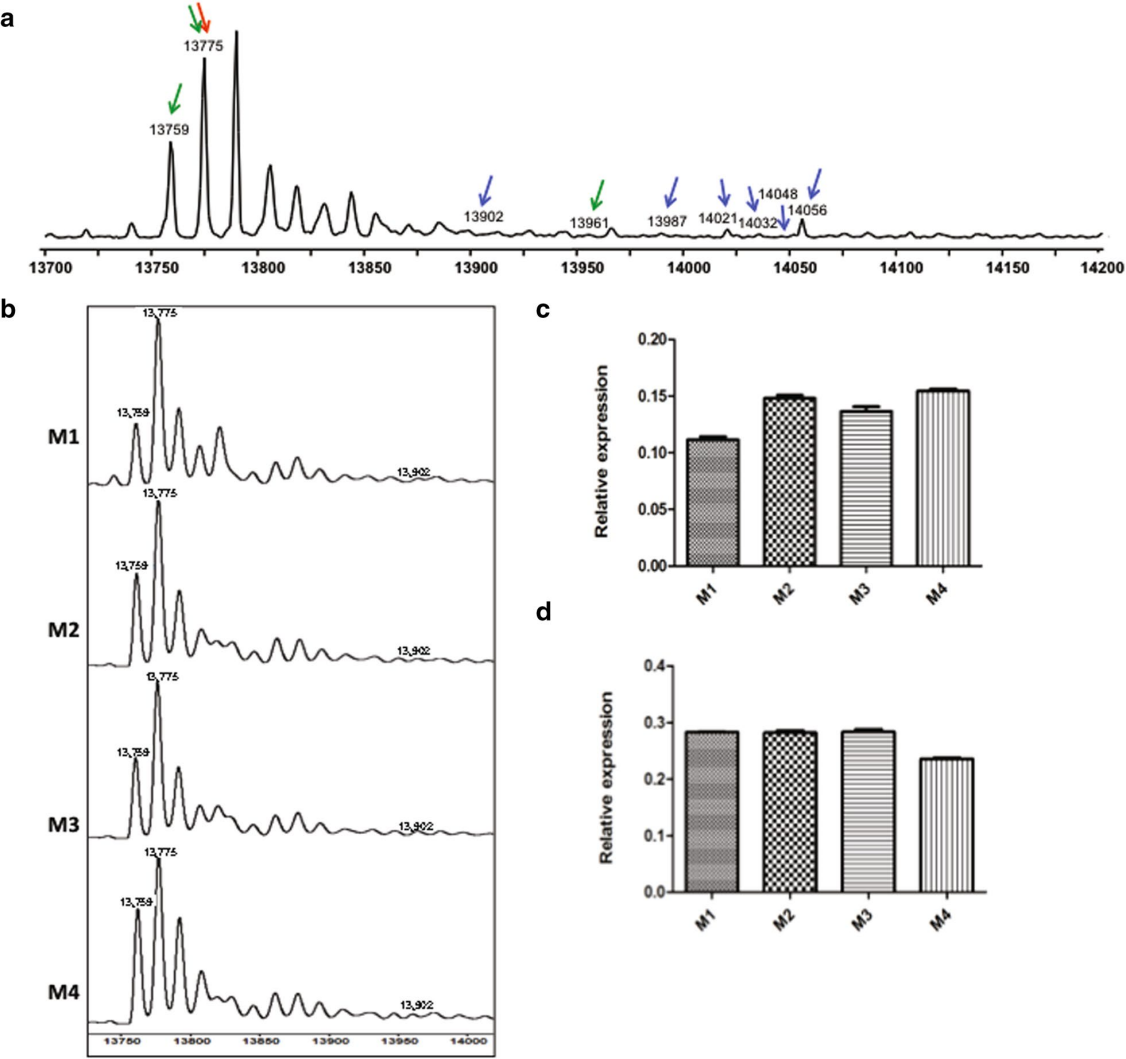
Histone H2B isoforms altered in CLL. **a** A representative histone H2B spectrum. The peaks that are significantly changed in CLL, bladder and breast cancer cells are highlighted with arrows (CLL-*green*, bladder-*red* and breast-*blue*). **b** A spectrum showing a change in some of the H2B isoforms in breast cancer. The isoforms that are most significantly changed have their respective molecular weight written on top of the peak. **c**, **d** Histograms representing the relative abundance of the peaks with molecular weight ~13,759 and ~13,775 Da. The relative abundance of each isoform has been determined as described in Fig. 2b

Replication-dependent histone H2B genes also encode for a large family of distinct polypeptides, with 22 genes yielding a total of 18 proteins (Additional file 1: Table S2). A typical spectrum of histone H2B from healthy CD19+ B cells is shown in Fig. 3a. While a number H2B species displayed altered abundance in the CLL patient samples, these species were all very low in abundance and are also highly modified (Table 1).

#### Bladder and breast cancer models

**Fig. 4:**
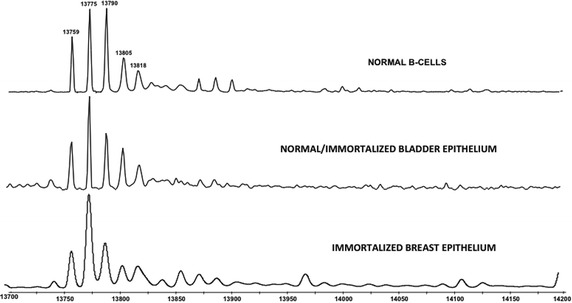
Profile of the replication-dependent H2B isoforms in our different model systems. LC/MS spectrum of histone H2B isolated from normal B-cells, hTERT immortalized bladder epithelium and immortalized breast epithelium (as indicated)

In the bladder cancer cell lines, there was a progressive increase in the H2B species with a M.W. of 13,775 Da, which represents the di-methylated form of H2B 1C, as the cells became more aggressive (Additional file 1: Table S6a), although it did not reach statistical significance. However, in contrast to the CLL patient samples, there were changes in the relative abundance of several of the most abundant replication-dependent H2B species in the breast cancer cell culture models (Table 2). Interestingly, the histone H2B profile in the breast cancer cells also showed a change in the abundance of methylated forms of H2B 1C (Table 2). In contrast to bladder cancer, the di-methylated form decreased in the highly malignant M4 cells. On the other hand, the mono-methylated form showed a concomitant increase, as the breast cancer cells became malignant (Fig. 3b–d; Table 2). Also of note is that the profile of the replication-dependent H2B in the normal bladder and breast epithelium differs dramatically from the profile of the normal B cells (Fig. 4). This suggests that there may be important tissue-specific changes in the complement of this histone.

### Global changes in histone H3 species in leukemic and cancer cells

**Fig. 5:**
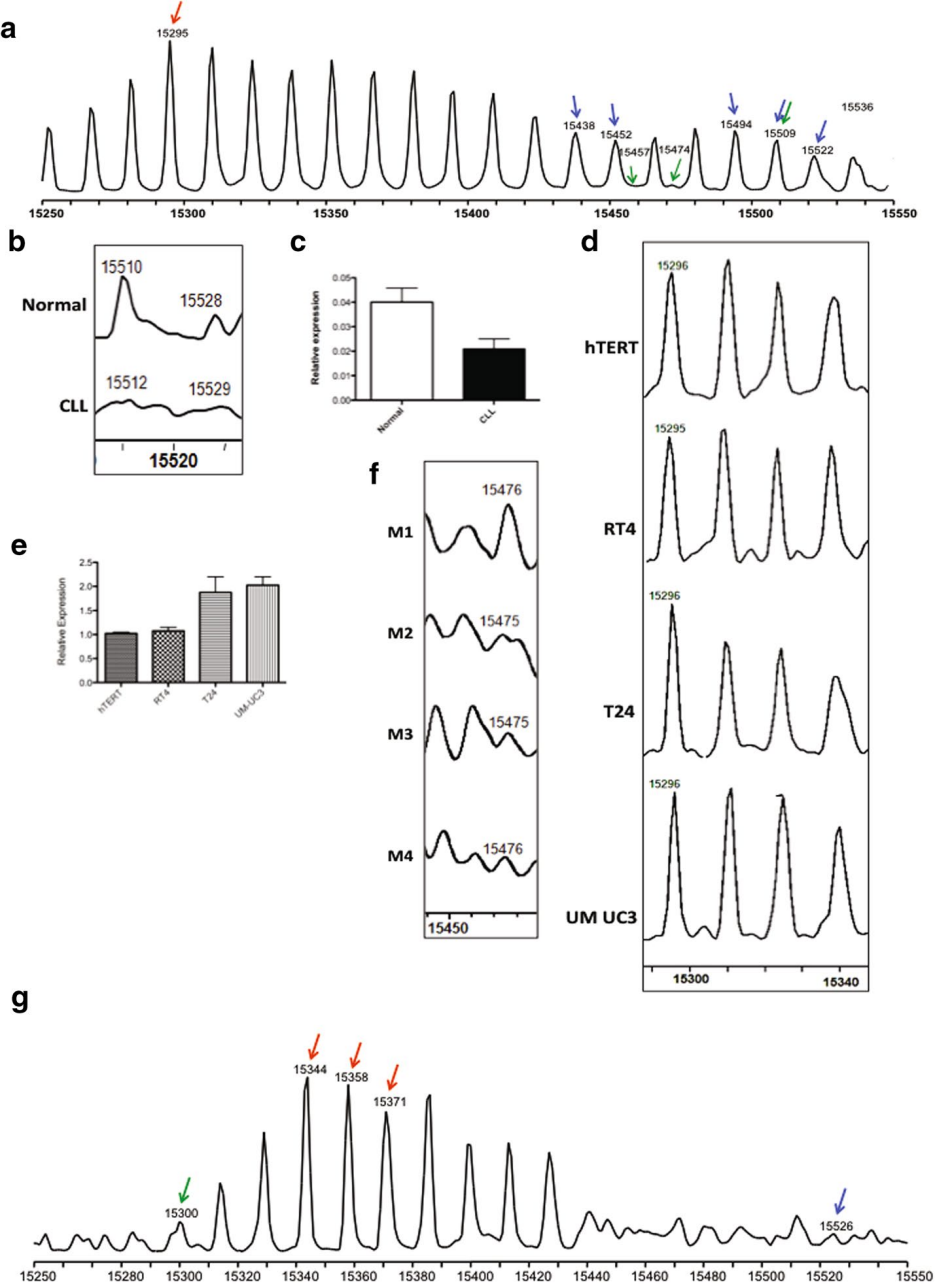
Histone H3 isoforms altered in leukemic and cancer cells. **a** A representative histone H3 peak 1 LC/MS spectrum. The peaks that are significantly changed in CLL, bladder and breast cancer cells are highlighted with arrows (CLL-*green*, bladder-*red* and breast-*blue*). **b**, **c** Representative spectrum and histogram, respectively, showing the relative decrease in abundance of the peak with molecular weight ~15,510 Da in CLL B cells. The relative abundance of each isoform has been determined as described in Fig. 2b. **d**, **e** Representative spectrum and histogram, respectively, showing the relative abundance of the peak with molecular weight ~15,296 Da in bladder cancer. **f** A spectrum representing the decreased abundance of the peak with molecular weight ~15,476 Da in breast cancer cells. **g** A representative histone H3 peak 2 LC/MS spectrum. The peaks are labeled as described above

The global analysis of histone H3 is complicated by a number of factors. First, under the LC conditions used, H3 elutes in two distinct peaks (data not shown). Hence, spectra from these peaks have been analyzed separately. These populations of histone H3 will be referred to as peak 1 (retention time ~36 min) and peak 2 (retention time ~42 min) based on the order in which they elute from a C18 column. Second, histone H3 is the most highly modified of the core histones. In particular, the high degree of methylation on histone H3 leads to the appearance of numerous peaks that are separated by 14 Da (Fig. 5a, g).

### Histone H3 Peak 1

#### Chronic lymphocytic leukemia

A representative spectrum of peak 1 histone H3 is shown in Fig. 5a. We observed down-regulation of several peak 1 histone H3 species in the CLL tissue samples (Table 1). Intriguingly, while changes in H3 acetylation and methylation have been linked to a number of cancers, the most significantly down-regulated species in peak 1 were also phosphorylated (Fig. 5b, c) [1–3, 5–7, 21].

#### Bladder and breast cancer models

Comparison of the peak 1 histone H3 profiles in the series of bladder cancer cell lines indicated that these histones were largely unchanged. The only species that increased in abundance with cancer aggression were unmodified and monomethylated versions of H3.2. However their adjusted p-values did not reach statistical significance. A few of the peak 1 histone H3 species changed in the breast cancer cell lines. The most significantly altered species was a decreased level of the mono-methylated, mono-phosphorylated form of the lone histone H3 isoform found in histone cluster 3 (H3/3 or H3.1t, Table 2). Intriguingly, the abundance of this species, which was originally thought to be testis-specific, was also significantly down-regulated in the CLL patient samples (Table 1) [22, 23]. Some of the species that are altered in bladder and breast cancer are shown in Fig. 5d–f.

### Histone H3 Peak 2

#### Chronic lymphocytic leukemia

The peak 2 population of histone H3 contained an entirely different complement of species and, based solely on the masses observed, there is no obvious explanation of why this pool of molecules has a distinct LC elution profile (Fig. 5g). When comparing peak 2 H3 from healthy and CLL B cells, many species showed a statistically significant change in abundance (Table 1). Notably, many phosphorylated forms of H3.1t with differing degrees of methylation were altered.

#### Bladder and breast cancer models

As observed with peak 1, the histone H3 pool from peak 2 was also not largely different in either bladder or breast cancer cells. The only species that showed a statistically significant change in breast cancer cells were the highly modified forms of H3.1 (Table 2). In the bladder cancer cell lines, only the ummodifed form of histone H3.2 showed an increased level in aggressive cell lines, though it did not reach statistical significance (Additional file 1: Table S6a).

### Global changes in histone H4 species in leukemic and cancer cells

#### Chronic lymphocytic leukemia

Among the core histones, histone H4 contains the least variation. There are no replication-independent forms of histone H4 and of the 14 replication-dependent H4 genes, 12 encode identical protein products. The other two genes encode minor, but detectable, forms of H4 (Additional file 1: Table S4). Similar to the situation with H3, comparison of healthy and CLL B cells showed that H4 levels were mostly unchanged, with 11 of the low abundance H4 species showing a statistically significant change (Table 1).

#### Bladder and breast cancer models

**Fig. 6:**
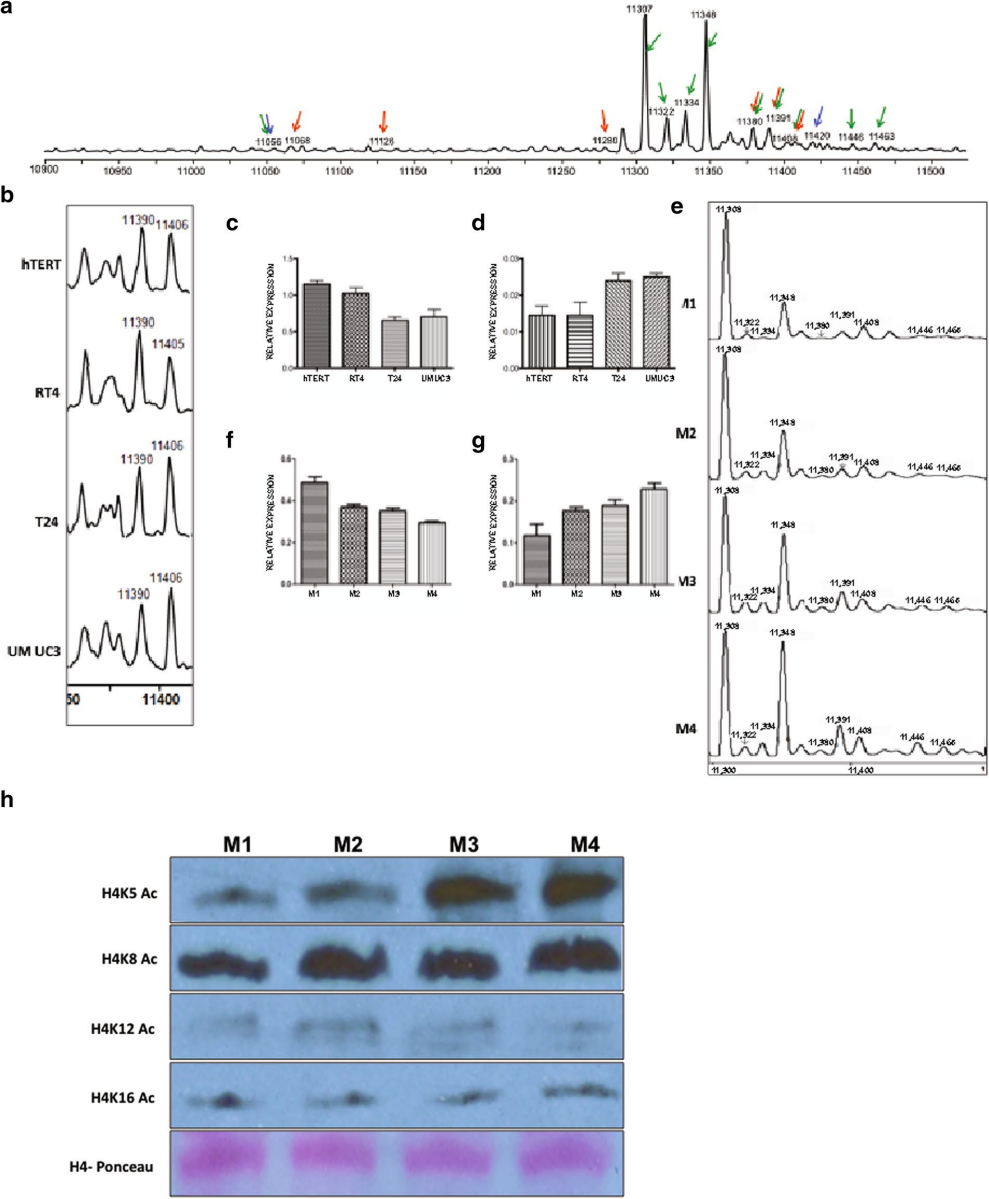
Histone H4 isoforms altered in leukemic and cancer cells. **a** A representative histone H4 LC/MS spectrum. The peaks that are significantly changed in CLL, bladder and breast cancer cells are highlighted with arrows (CLL-*green*, bladder-*red* and breast-*blue*). **b**–**d** Representative spectrum (**b**) and histograms representing the relative abundance of the peaks with molecular weight ~11,390 Da (**c**) and ~11,406 Da (**d**) in bladder cancer cells. The relative abundance of each isoform has been determined as described in Fig. 2b. **e**–**g** Representative spectra (**e**) and histograms representing the relative abundance of the peaks with molecular weight ~11,306 Da (**f**) and ~11,348 Da (**g**) respectively in breast cancer cells. **h** Western blot analysis of histones isolated from the M1–M4 cell lines (as indicated). Western blots were probed with antibodies recognizing the indicated sites of acetylation on the NH_2_-terminal tail of histone H4. The *bottom panel* shows the Ponceau S staining of the blot prior to antibody incubation

Relative to the immortalized normal bladder epithelial cells, there was some alteration of low abundance H4 species in the bladder cancer cells (Additional file 1: Table S6a). Some of the isoforms that changed in abundance are shown in Fig. 6b–d. However, the M1 to M4 breast cancer cell lines demonstrated dramatic changes in high abundance peaks (Fig. 6e–g; Table 2). Most apparent was the increased abundance of the di-methylated and mono-acetylated form of H4, with a molecular weight of 11,348 (Fig. 6e). There were also increased amounts of forms of H4 that contained higher levels of acetylation (Table 2). As changes in the acetylation state of H4 lysine 16 has been shown to be a hallmark of many cancers, we sought to identify which residue(s) were becoming acetylated as the breast cancer cell lines became more metastatic. Western blot analysis using antibodies recognizing acetylation at the 4 sites of acetylation on the NH_2_-terminal tail of H4 indicated that the increased acetylation observed is primarily due to the modification of lysine 5 (Fig. 6h). Notably, the acetylation of lysine 16 does not appreciably change across these cell lines. While H4 lysine 5 acetylation clearly correlated with malignant phenotype of the breast cancer cell lines, it will be important to determine whether this modification plays a causative role.

## Discussion

Previous global analyses of histones in cancer have focused on the post-translational modification patterns of histones H3 and H4. These studies have identified several specific modifications that strongly correlate with tumorigenesis. In particular, decreases in the levels of H4 lysine 16 acetylation and lysine 20 methylation and H3 lysine 18 acetylation, lysine 4 methylation and lysine 9 methylation have been seen in a large number of tumor types and tumor cell models [1, 5, 6, 8, 21, 24–27]. However, we have found that the abundance of the post-translationally modified forms of H3 and H4 are quite stable across a large number of CLL patient samples with most of the changes limited to relatively low abundance species. This suggests that alterations of H3 and H4 are not universally hallmarks of malignant transformation.

The paucity of changes in high abundance species observed in CLL patient samples may have a number of explanations. From a biological perspective, CLL may be distinct from the limited range of other malignancies that have been explored, such as prostate and lung cancer. There may also be technical reasons that could prevent our analyses from detecting such changes. Our study has relied on mass spectrometry rather than immunological methods. This allows us to measure the relative abundance of a specifically modified histone species but does not allow us to localize the modifications to a specific residue. Hence, if there were a decrease in the level of H4 lysine 16 acetylation in the CLL cells but a concomitant increase in the acetylation of another lysine residue on H4, there would be no net change in acetylation detected.

There was a more dramatic change in the profile of histone H4 in the breast cancer cell lines. There was a much higher level of the 11,349 Da species in the M4 cells relative to the M1 cells. Based on this molecular weight, this species is likely to be the di-methylated/mono-acetylated form of H4. Based on previous analyses, the majority of this species typically contains H4 lysine 20 dimethylation and lysine 16 acetylation [4]. Progressive increases in this methylated/acetylated species of H4 across the spectrum of aggressiveness of this series of breast cancer cell lines are contrary to immunohistochemical analysis of breast tumor samples [6]. However, while we appreciate that no cell culture system fully recapitulates the complexity of human disease and that there are differences in the information obtained between mass spectrometry and immunohistochemical techniques, these differences might give us insight into the behavior of different subtypes of breast cancer.

One of the most important reasons for the use of mass spectrometry to characterize the histone proteome is that it is an unbiased approach that does not rely on the existence of modification-specific antibodies. As there are relatively few immunological reagents specific for the characterization of post-translational modifications on histones H2A and H2B, our analysis is the most comprehensive study of these histones in cancer, to date. Our results indicate that the patterns of histones H2A and H2B found in both primary tumor tissue samples and cancer cell line models are highly dynamic. These changes were observed both between healthy and cancer cells and between different types of cancer cells. For example, the pattern of H2B observed in B cells was very different from that observed in the solid tumor cell lines. This suggests the possibility that there may be tissue specific variations in the patterns of histone H2A and H2B species.

The dynamic nature of histone H2A is not limited to its pattern of post-translational modifications. We also detect a wide range of variation in the abundance of specific replication-dependent isoforms of histone H2A. While the functional analysis of these distinct replication-dependent H2A isoforms is only beginning, the observation that their abundances are altered in primary tumor tissue supports the intriguing possibility that they may encode functionally distinct molecules [16, 17].

The most clinically relevant species in the histone complement of CLL cells may be that with a molecular mass of 14,063 Da. The CLL patient samples could be divided into two groups based on the presence or absence of this species. Those patients with a detectable level of this species had a significantly shorter time to treatment; its prognostic relevance will need to be verified in an independent dataset. Additionally, identification of the molecular make-up of this species will be needed to determine whether this form of histone H2A plays a functional role in disease progression.

## Conclusions

We have performed a comprehensive proteomic analysis of the core histones from CD19+ B cells from healthy individuals and the malignant counterpart in CLL patient samples as well as from bladder and breast cancer cell line models. Using LC/MS, we have quantified the relative abundance of every detectable histone peak to identify histone species whose abundance correlates with the presence or severity of disease. Our results identified interesting candidates whose levels show a significant correlation to disease aggression and can be explored further. In addition, we demonstrated that the abundance of the vast majority of histone species do not significantly change in primary tumor samples and cell model systems suggesting that at least some malignancies are not accompanied by large-scale alterations in the global histone proteome.

## Methods

### Cell lines and culture conditions

Peripheral blood was obtained from patients diagnosed with CLL by NCI 1996 criteria (Ref: Cheson et al., Blood. 1996; 87(12):4990–7) or from healthy individuals under protocols approved by the Ohio State University Institutional Review Board, according to the Declaration of Helsinki. Clinical status of CLL patients varied, but all CLL samples contained elevated lymphocyte count (>30,000 CD19+ cells/uL). CD19+ cells were obtained by negative selection using reagents from StemCell Technologies (Vancouver BC). M1–M4 cells were a gift from Dr. Tsonwin Hai. The cells were grown in DMEM/F-12 with 5 % horse serum (Invitrogen), 0.029 M sodium bicarbonate (Sigma), 10 mM HEPES (Sigma), 10 µg/ml insulin (Sigma), 10 ng/ml EGF (Millipore), 0.5 µg/ml hydrocortisone (Sigma), 100 ng/ml cholera toxin (Calbiochem) and 1 % penicillin/streptomycin (Sigma). hTERT cells are immortalized breast epithelial cells, RT4 cells are transformed but non-malignant, T24 cells are malignant but non-metastatic and UM-UC-3 cells are malignant and highly metastatic. The cells were grown in DMEM, supplemented with 10 % FBS and 1 % penicillin/streptomycin (Sigma). All the cell types as described above were incubated at 37 °C in a humidified atmosphere with 5 % CO_2_ and 95 % air.

### Mass spectrometry

Histones were prepared with standard acid extraction procedure as described [28]. Extracted histones were subjected to LC–MS analysis. Characterization was performed by HPLC separation (Dionex, Waltham, MA) in line with either a MicroMass Q-TOF (MicroMass, Milford, MA) or AmaZon ETD (Burker, Billerica, MA) mass spectrometers.

For the Q-TOF MS analysis, approximately 20 μg of extracted histones were HPLC separated on a Dionex Ultimate 3000 capillary/nano HPLC (Dionex, Waltham, MA) fitted with a 1.0 × 150 mm C18 column (Discovery Bio wide pore C18 column, 5 μm, 300 Å, Supelco, USA) as described previously by Wang et al. [29]. Mobile phase A contained 0.05 % TFA (aq) (Pierce, Rockford, IL). Mobile phase B contained 0.05 % TFA in acetonitrile (EMD Millipore, Billerica, MA). Initial conditions were 20 % B, with linear increases to 30 % at 2 min, 35 % at 10 min, 50 % B at 30 min, 60 % at 35 min and 95 % at 36 min. The column was washed at 95 % B for 4 min. Column equilibration was conducted at the initial conditions for 15 min. Total run time was 55 min. Blank injections of the same gradient were made between each sample injection. The mass range (500-2500 m/z) was scanned every 0.6 s in positive ion mode. A representative raw spectrum for each histone variant has been provided in Additional file 2: Fig. S2. Savitzky Golay smoothing (smooth window ± 3 channels, 2 cycles), mass deconvolution (MaxEnt algorithm, 1.0 Da/channel resolution, 0.750 Da uniform Gaussian width at half height damage model, 33 % left right minimum intensity ratios, iterate to convergence) and mass analysis were conducted using the MassLynx software 4.0 (Waters Corp., Milford, MA).

For AmaZon ETD analysis, approximately 100 ng of extracted histones were subjected to a modified LC–MS method described by You et al. [30]. HPLC separation was performed on a Dionex Ultimate 3000 capillary/nano HPLC (Dionex, Waltham, MA) fitted with a 0.3 × 150 mm Magic C8 column (5 μm, 300 Å, Michrom Bioresources, Auburn, CA). Mobile phases were HPLC water (J.T. Baker, Center Valley, PA) and acetonitrile (EMD Millipore, Billerica, MA) each supplemented with 0.5 % formic acid (v/v, Acros Organics, Waltham, MA). Initial conditions were 20 % B held for 5 min, the gradient increased to 30 % B at 25 min and 35 % B at 65 min each with a convex curvature of 2. From 65 to 78 min, the gradient increased from 35 to 48 % B with a convex curvature of 4. Column equilibration at the initial conditions was conducted 12 min. Total run time was 90 min. Blank injections of the same gradient were made between each sample injection. A 500–2500 m/z mass range was scanned every 0.2 s in positive ion mode. A representative raw spectrum for each histone variant has been provided in Additional file 2: Fig. S3. Savitzky Golay smoothing (3 m/z width, 2 smooth cycles), Maximum Entropy deconvolution (data point spacing auto m/z, instrument peak width 0.3, resolution normal) and data analysis was conducted using the Bruker ESI Compass Data Analysis 1.3 software for AmaZon (ver 4.0 SP4, Bruker, Billerica, MA).

For quantitation of an isoform, the peak intensities of all the isoforms corresponding to a particular histone were pooled. The abundance of a particular isoform was then calculated relative to the pooled intensity of all detectable forms of that histone. A given peak was therefore quantitated relative to the total amount of histone present in the sample. The same approach was followed for the results derived from both the Q-Tof and Amazon mass spectrometers.

### Statistical analysis

For each histone, the expression for a particular species was measured relative to the total expression across all species. The nonparametric Wilcoxon rank sum test was used to compare (1) the proportion expressed between normal donor and CLL patient samples and (2) the proportion expressed between patients samples with CLL and Zap70 positive (>20 %) versus negative protein. In these patients, time to treatment was measured from the date of diagnosis until the date of first treatment, censoring those who had not yet started treatment at the date of last follow-up. Associations between expression of a species (using median expression to group patients as high and low expressers) and time to treatment were evaluated using the score test from proportional hazards models; estimates of hazard ratios with confidence intervals were estimated from the model. Difference in time to treatment between high and low expressers for a particular species is shown graphically in a Kaplan–Meier plot. All tests are 2-sided and statistical significance was declared when the false discovery rate (FDR) adjusted p-value was less than 0.05, allowing for 5 % of the significant tests to be false positives. If none of the variants are statistically significant when applying the multiple testing corrections then it may be possible that the findings are false positives. However, to provide complete information, all the isoforms have been ordered for each analysis from smallest to largest p-value without any correction, with results corresponding to the smallest p-value as most reliable. The results have been sorted as separate tables based on if the isoforms were significant at least at the raw p-value levels (Additional file 1: Tables S5a, S6a and S7a) or non-significant even at raw p-value levels (Additional file 1: Tables S5b, S6b and S7b). A few of the isoforms had an FDR < 0.05, but were unidentified and therefore omitted from the study. The differences in expression across breast and bladder cell lines with varying degrees of aggressiveness were screened using one-way ANOVA. The cell lines were analyzed in triplicates and error bars represent the standard error of mean between the samples. All statistical analyses were conducted using the SAS (University Edition) statistical software package (SAS Institute Inc., Cary, NC).

Western Blotting: The M1–M4 cell lines were grown as described above. The cells were seeded in equal numbers, harvested after 48 h and histone preparation was carried out as described elsewhere (ref 29). The histones were quantitated using Bradford assay and about 20 μg of protein was used for western analysis. All the antibodies used in the analysis were purchased commercially (Abcam, Cambridge, MA). Briefly, the samples were loaded on 12.5 % polyacrylamide gels, transferred to PVDF membranes and Ponceau stained. The images were taken to confirm the equal loading followed by blocking in 5 % skimmed milk. The blots were the incubated in respective primary and HRP-conjugated secondary antibodies (G E Healthcare, UK) in 1:1000 and 1:5000 dilutions respectively. After each incubation, the blots were washed three times with TBST and finally developed using Pierce™ ECL Western Blotting Substrate (Life Technologies). The blots were then exposed to X-ray film for 60 s (Denville Scientific Inc, South Plainfield, NJ). The process was repeated three times using different passages of cell lines to ensure reproducibility.

## Additional files

Additional file 1. 12 tables listing all of the core histone variants and their molecular weights. Additional tables list the quantitation of all observable core histone isoforms in the samples analyzed. Additional file 2:
**Figure S1.** A schematic diagram of the method used to quantitate histone proteomics in tumor samples. **Figures S2, S3.** Examples of raw spectra.
