# Supplementary material for: Proteomic profiling identifies specific histone species associated with leukemic and cancer cells
Source: Clin Proteomics. 2015 Aug 27;12(1):22. doi: 10.1186/s12014-015-9095-4 (PMC4551702; doi:10.1186/s12014-015-9095-4)
Supplement: Additional file 1. — 12 tables listing all of the core histone variants and their molecular weights. Additional tables list the quantitation of all observable core histone isoforms in the samples analyzed. [file 12014_2015_9095_MOESM1_ESM.docx]

**SUPPLEMENTARY TABLES**

**Table 1- Histone H2A isoforms with the corresponding protein name and molecular weight**

**Table 2- Histone H2B isoforms with the corresponding protein name and molecular weight**

**Table 3- Histone H3 isoforms with the corresponding protein name and molecular weight**

**Table 4- Histone H4 isoforms with the corresponding protein name and molecular weight**

**Table 5a- Histone isoforms that are significantly changed in CLL at raw p-value levels**

**Table 5b- Histone isoforms that are not significantly changed in CLL at raw p-value levels**

Table 5b- contd…

Table 5b- contd…

**Table 6a- Histone isoforms that are significantly changed in bladder cancer at raw p-value level**

**Table 6b- Histone isoforms that are not significantly changed in bladder cancer**

**Table 6b- Continued……**

**Table 6b- Continued……**

**Table 6b- Continued……**

**Table 7a- Histone isoforms that are significantly changed in breast cancer at raw p-value level**

**Table 7b- Histone isoforms that are not significantly changed in breast cancer**

**Table 7b- Continued……**

**Table 8- Correlation of Histone isoforms with Zap-70**

| **Variant** | **Fragment** | **Raw P83** | **FDR83** | **Raw P64** | **FDR64** |
| --- | --- | --- | --- | --- | --- |
| H3_1 | 15267 | 0.0032 | 0.3234 | 0.0013 | 0.1617 |
| H3_2 | 15371 | 0.0042 | 0.3234 | 0.0014 | 0.1617 |
| H3_1 | 15281 | 0.0070 | 0.3234 | 0.0023 | 0.1771 |
| H4 | 11014 | 0.0020 | 0.3234 | 0.0082 | 0.4179 |
| H3_2 | 15400 | 0.0587 | 0.5594 | 0.0122 | 0.4179 |
| H2A | 14033 | 0.0355 | 0.5164 | 0.0149 | 0.4179 |
| H3_2 | 15344 | 0.0618 | 0.5594 | 0.0163 | 0.4179 |
| H3_1 | 15547 | 0.1801 | 0.671 | 0.0178 | 0.4179 |
| H3_1 | 15404 | 0.0362 | 0.5164 | 0.0186 | 0.4179 |
| H4 | 11002 | 0.0062 | 0.3234 | 0.0193 | 0.4179 |
| H2A | 14004 | 0.1057 | 0.621 | 0.0200 | 0.4179 |
| H3_2 | 15414 | 0.0199 | 0.5164 | 0.0225 | 0.4179 |
| H3_1 | 15332 | 0.1084 | 0.621 | 0.0263 | 0.4179 |
| H3_2 | 15428 | 0.2305 | 0.7735 | 0.0282 | 0.4179 |
| H3_2 | 15485 | 0.0424 | 0.5439 | 0.0295 | 0.4179 |
| H3_2 | 15358 | 0.0369 | 0.5164 | 0.0336 | 0.4179 |
| H2A | 13932 | 0.0296 | 0.5164 | 0.0337 | 0.4179 |
| H2A | 13847 | 0.0296 | 0.5164 | 0.0360 | 0.4179 |
| H3_1 | 15296 | 0.1252 | 0.6261 | 0.0366 | 0.4179 |
| H2A | 14103 | 0.0105 | 0.4043 | 0.0394 | 0.4179 |
| H3_2 | 15442 | 0.0320 | 0.5164 | 0.0398 | 0.4179 |
| H3_2 | 15386 | 0.1091 | 0.621 | 0.0398 | 0.4179 |
| H3_1 | 15310 | 0.1116 | 0.621 | 0.0451 | 0.4447 |
| H4 | 11005 | 0.1125 | 0.621 | 0.0462 | 0.4447 |
| H4 | 11438 | 0.2584 | 0.7752 | 0.0503 | 0.4648 |
| H3_1 | 15533 | 0.1781 | 0.671 | 0.0605 | 0.497 |
| H4 | 10965 | 0.1465 | 0.6552 | 0.0611 | 0.497 |
| H3_2 | 15329 | 0.0380 | 0.5164 | 0.0622 | 0.497 |
| H2B | 13987 | 0.0478 | 0.5439 | 0.0624 | 0.497 |
| H2B | 13928 | 0.0635 | 0.5594 | 0.0654 | 0.5036 |
| H4 | 11542 | 0.0862 | 0.621 | 0.0703 | 0.515 |
| H3_2 | 15298 | 0.0651 | 0.5594 | 0.0726 | 0.515 |
| H4 | 11039 | 0.0300 | 0.5164 | 0.0756 | 0.515 |
| H2B | 13747 | 0.0476 | 0.5439 | 0.0758 | 0.515 |
| H3_2 | 15499 | 0.1552 | 0.6552 | 0.0808 | 0.5219 |
| H2A | 14184 | 0.1703 | 0.6668 | 0.0830 | 0.5219 |
| H3_2 | 15294 | 0.0515 | 0.5439 | 0.0836 | 0.5219 |
| H3_1 | 15274 | 0.1846 | 0.6769 | 0.0861 | 0.5234 |
| H2B | 13957 | 0.0669 | 0.5594 | 0.0903 | 0.5273 |
| H3_2 | 15457 | 0.2366 | 0.7735 | 0.0913 | 0.5273 |
| H2B | 13790 | 0.1732 | 0.6668 | 0.0947 | 0.5336 |
| H2B | 13944 | 0.0264 | 0.5164 | 0.0990 | 0.5445 |
| H3_1 | 15498 | 0.0833 | 0.621 | 0.1053 | 0.5657 |
| H3_2 | 15261 | 0.1177 | 0.6261 | 0.1181 | 0.6155 |
| H3_2 | 15506 | 0.2456 | 0.7735 | 0.1199 | 0.6155 |
| H4 | 11433 | 0.2347 | 0.7735 | 0.1370 | 0.6728 |
| H2B | 13877 | 0.1682 | 0.6668 | 0.1391 | 0.6728 |
| H3_2 | 15468 | 0.3494 | 0.7991 | 0.1398 | 0.6728 |
| H3_1 | 15339 | 0.5721 | 0.9489 | 0.1475 | 0.6954 |
| H4 | 11019 | 0.1620 | 0.6668 | 0.1566 | 0.705 |
| H3_1 | 15527 | 0.1129 | 0.621 | 0.1577 | 0.705 |
| H2A | 13817 | 0.4160 | 0.8657 | 0.1587 | 0.705 |
| H3_1 | 15390 | 0.0890 | 0.621 | 0.1743 | 0.731 |
| H2B | 14032 | 0.3880 | 0.8455 | 0.1746 | 0.731 |
| H3_1 | 15317 | 0.1508 | 0.6552 | 0.1834 | 0.731 |
| H3_2 | 15314 | 0.1281 | 0.6261 | 0.1845 | 0.731 |
| H3_2 | 15493 | 0.0678 | 0.5594 | 0.1879 | 0.731 |
| H4 | 11373 | 0.3258 | 0.7991 | 0.1880 | 0.731 |
| H2B | 13819 | 0.1675 | 0.6668 | 0.1882 | 0.731 |
| H2A | 14096 | 0.0881 | 0.621 | 0.1904 | 0.731 |
| H2B | 14002 | 0.3211 | 0.7991 | 0.1950 | 0.731 |
| H3_1 | 15367 | 0.9540 | 0.9996 | 0.1962 | 0.731 |
| H4 | 11322 | 0.1889 | 0.6818 | 0.2018 | 0.7399 |
| H4 | 11250 | 0.1560 | 0.6552 | 0.2054 | 0.7414 |
| H2B | 13759 | 0.1943 | 0.6905 | 0.2161 | 0.7599 |
| H4 | 11048 | 0.4022 | 0.8549 | 0.2202 | 0.7599 |
| H2A | 13911 | 0.2412 | 0.7735 | 0.2204 | 0.7599 |
| H3_1 | 15510 | 0.2698 | 0.7763 | 0.2327 | 0.7706 |
| H3_2 | 15534 | 0.2869 | 0.7797 | 0.2328 | 0.7706 |
| H3_2 | 15449 | 0.2384 | 0.7735 | 0.2335 | 0.7706 |
| H4 | 11511 | 0.3810 | 0.8455 | 0.2381 | 0.7747 |
| H2B | 14095 | 0.1021 | 0.621 | 0.2418 | 0.7758 |
| H3_1 | 15448 | 0.6079 | 0.9531 | 0.2536 | 0.8025 |
| H3_1 | 15325 | 0.5643 | 0.9489 | 0.2632 | 0.8216 |
| H4 | 11519 | 0.4812 | 0.9233 | 0.2743 | 0.8329 |
| H2B | 13805 | 0.3465 | 0.7991 | 0.2806 | 0.8329 |
| H3_2 | 15461 | 0.4526 | 0.9091 | 0.2822 | 0.8329 |
| H2B | 13921 | 0.5341 | 0.9276 | 0.2862 | 0.8329 |
| H2A | 13899 | 0.1339 | 0.6312 | 0.2897 | 0.8329 |
| H4 | 11484 | 0.2228 | 0.7682 | 0.2919 | 0.8329 |
| H3_1 | 15515 | 0.0518 | 0.5439 | 0.2994 | 0.8329 |
| H2A | 14168 | 0.5124 | 0.9233 | 0.3013 | 0.8329 |
| H4 | 11488 | 0.6924 | 0.971 | 0.3041 | 0.8329 |
| H3_2 | 15538 | 0.3471 | 0.7991 | 0.3060 | 0.8329 |
| H2B | 13898 | 0.2725 | 0.7763 | 0.3086 | 0.8329 |
| H3_2 | 15472 | 0.3151 | 0.7991 | 0.3101 | 0.8329 |
| H3_1 | 15489 | 0.2017 | 0.706 | 0.3185 | 0.8361 |
| H3_2 | 15283 | 0.2968 | 0.7851 | 0.3185 | 0.8361 |
| H3_1 | 15424 | 0.3654 | 0.8195 | 0.3227 | 0.8376 |
| H2A | 14147 | 0.3343 | 0.7991 | 0.3353 | 0.8499 |
| H3_2 | 15478 | 0.5633 | 0.9489 | 0.3356 | 0.8499 |
| H3_1 | 15537 | 0.1301 | 0.6261 | 0.3385 | 0.8499 |
| H4 | 11043 | 0.2478 | 0.7735 | 0.3482 | 0.8649 |
| H3_1 | 15494 | 0.9259 | 0.9996 | 0.3572 | 0.8654 |
| H3_1 | 15382 | 0.9908 | 1 | 0.3624 | 0.8654 |
| H3_1 | 15376 | 0.5889 | 0.9513 | 0.3675 | 0.8654 |
| H2A | 14083 | 0.1202 | 0.6261 | 0.3698 | 0.8654 |
| H4 | 11516 | 0.6792 | 0.971 | 0.3698 | 0.8654 |
| H2B | 13831 | 0.1224 | 0.6261 | 0.3709 | 0.8654 |
| H3_1 | 15457 | 0.1401 | 0.6473 | 0.3853 | 0.8839 |
| H2B | 13971 | 0.1064 | 0.621 | 0.3894 | 0.8839 |
| H2A | 14063 | 0.3025 | 0.7851 | 0.3903 | 0.8839 |
| H4 | 11497 | 0.3868 | 0.8455 | 0.4146 | 0.9278 |
| H3_1 | 15434 | 0.3245 | 0.7991 | 0.4177 | 0.9278 |
| H3_2 | 15279 | 0.6900 | 0.971 | 0.4237 | 0.9321 |
| H4 | 11348 | 0.4432 | 0.906 | 0.4298 | 0.9366 |
| H2A | 13916 | 0.2571 | 0.7752 | 0.4381 | 0.9458 |
| H2B | 13775 | 0.0317 | 0.5164 | 0.4453 | 0.9517 |
| H2A | 14157 | 0.6728 | 0.971 | 0.4527 | 0.9517 |
| H4 | 11446 | 0.9250 | 0.9996 | 0.4532 | 0.9517 |
| H2B | 14048 | 0.5733 | 0.9489 | 0.4627 | 0.9629 |
| H2B | 13839 | 0.0823 | 0.621 | 0.4739 | 0.9651 |
| H2B | 13881 | 0.7952 | 0.9926 | 0.4757 | 0.9651 |
| H2B | 13952 | 0.6148 | 0.9531 | 0.4806 | 0.9651 |
| H3_1 | 15353 | 0.9265 | 0.9996 | 0.4823 | 0.9651 |
| H3_2 | 15548 | 0.4354 | 0.898 | 0.5012 | 0.9651 |
| H3_1 | 15444 | 0.5257 | 0.9276 | 0.503 | 0.9651 |
| H2A | 14132 | 0.6742 | 0.971 | 0.5044 | 0.9651 |
| H2B | 14083 | 0.6687 | 0.971 | 0.5205 | 0.9651 |
| H3_2 | 15519 | 0.2967 | 0.7851 | 0.5221 | 0.9651 |
| H3_1 | 15485 | 0.2742 | 0.7763 | 0.5223 | 0.9651 |
| H4 | 11384 | 0.4065 | 0.8549 | 0.5235 | 0.9651 |
| H3_2 | 15321 | 0.6582 | 0.971 | 0.5255 | 0.9651 |
| H2B | 13963 | 0.1057 | 0.621 | 0.5276 | 0.9651 |
| H2A | 13890 | 0.4814 | 0.9233 | 0.5392 | 0.9651 |
| H3_2 | 15392 | 0.9899 | 1 | 0.5480 | 0.9651 |
| H3_1 | 15522 | 0.4071 | 0.8549 | 0.5518 | 0.9651 |
| H3_2 | 15407 | 0.5137 | 0.9233 | 0.5717 | 0.9651 |
| H2B | 13888 | 0.9453 | 0.9996 | 0.5804 | 0.9651 |
| H2B | 13855 | 0.9490 | 0.9996 | 0.5805 | 0.9651 |
| H3_2 | 15365 | 0.8937 | 0.9996 | 0.5830 | 0.9651 |
| H3_1 | 15431 | 0.2769 | 0.7763 | 0.5835 | 0.9651 |
| H4 | 10921 | 0.7093 | 0.971 | 0.5847 | 0.9651 |
| H4 | 11461 | 0.3023 | 0.7851 | 0.5850 | 0.9651 |
| H3_1 | 15289 | 1.0000 | 1 | 0.5971 | 0.9651 |
| H4 | 11546 | 0.9749 | 1 | 0.5982 | 0.9651 |
| H2A | 14112 | 0.7077 | 0.971 | 0.6034 | 0.9651 |
| H3_1 | 15347 | 0.4979 | 0.9233 | 0.6086 | 0.9651 |
| H2A | 14046 | 0.7839 | 0.9926 | 0.6130 | 0.9651 |
| H3_2 | 15528 | 0.9346 | 0.9996 | 0.6172 | 0.9651 |
| H3_1 | 15543 | 0.9906 | 1 | 0.6195 | 0.9651 |
| H2A | 13906 | 0.6673 | 0.971 | 0.6320 | 0.9651 |
| H3_2 | 15514 | 0.8084 | 0.9926 | 0.6322 | 0.9651 |
| H2B | 13902 | 0.9700 | 1 | 0.6365 | 0.9651 |
| H2A | 13858 | 0.9013 | 0.9996 | 0.6400 | 0.9651 |
| H3_2 | 15543 | 0.5870 | 0.9513 | 0.6450 | 0.9651 |
| H4 | 11503 | 0.4926 | 0.9233 | 0.6451 | 0.9651 |
| H3_1 | 15438 | 0.7294 | 0.971 | 0.6457 | 0.9651 |
| H3_1 | 15461 | 0.5156 | 0.9233 | 0.6522 | 0.9651 |
| H2A | 14057 | 0.3465 | 0.7991 | 0.6612 | 0.9651 |
| H2A | 13987 | 0.4591 | 0.9139 | 0.6612 | 0.9651 |
| H2A | 13943 | 0.7513 | 0.9805 | 0.6613 | 0.9651 |
| H4 | 11378 | 0.9321 | 0.9996 | 0.6737 | 0.9651 |
| H3_2 | 15256 | 0.9812 | 1 | 0.6768 | 0.9651 |
| H3_1 | 15260 | 0.7270 | 0.971 | 0.6788 | 0.9651 |
| H3_2 | 15264 | 0.8832 | 0.9996 | 0.6813 | 0.9651 |
| H2B | 13844 | 0.8550 | 0.9996 | 0.6833 | 0.9651 |
| H3_1 | 15472 | 0.2823 | 0.7763 | 0.6901 | 0.9651 |
| H2B | 14092 | 0.2643 | 0.7763 | 0.6904 | 0.9651 |
| H2B | 13934 | 0.6872 | 0.971 | 0.6954 | 0.9651 |
| H4 | 11526 | 0.7400 | 0.9734 | 0.6978 | 0.9651 |
| H3_1 | 15303 | 0.6582 | 0.971 | 0.7031 | 0.9651 |
| H2A | 14025 | 0.1498 | 0.6552 | 0.7032 | 0.9651 |
| H3_1 | 15452 | 0.8896 | 0.9996 | 0.708 | 0.9651 |
| H4 | 11028 | 0.8713 | 0.9996 | 0.7119 | 0.9651 |
| H4 | 11056 | 0.4629 | 0.9139 | 0.7161 | 0.9651 |
| H3_2 | 15489 | 0.6832 | 0.971 | 0.7170 | 0.9651 |
| H2A | 13940 | 0.4973 | 0.9233 | 0.7197 | 0.9651 |
| H3_2 | 15306 | 0.9562 | 0.9996 | 0.7232 | 0.9651 |
| H4 | 11389 | 0.7231 | 0.971 | 0.7322 | 0.9651 |
| H3_2 | 15272 | 0.6490 | 0.971 | 0.7408 | 0.9651 |
| H2A | 14173 | 0.6074 | 0.9531 | 0.7411 | 0.9651 |
| H2B | 13873 | 0.9126 | 0.9996 | 0.7420 | 0.9651 |
| H4 | 10946 | 0.8533 | 0.9996 | 0.7507 | 0.9651 |
| H4 | 11473 | 0.9428 | 0.9996 | 0.7571 | 0.9651 |
| H3_2 | 15436 | 0.8034 | 0.9926 | 0.7587 | 0.9651 |
| H3_2 | 15303 | 0.7165 | 0.971 | 0.7601 | 0.9651 |
| H2A | 14018 | 0.3372 | 0.7991 | 0.7615 | 0.9651 |
| H4 | 11292 | 0.5098 | 0.9233 | 0.7622 | 0.9651 |
| H3_2 | 15351 | 0.5124 | 0.9233 | 0.7625 | 0.9651 |
| H2B | 13940 | 0.5338 | 0.9276 | 0.7668 | 0.9651 |
| H2A | 14197 | 0.5815 | 0.9513 | 0.7704 | 0.9651 |
| H2A | 13958 | 0.9417 | 0.9996 | 0.7718 | 0.9651 |
| H3_2 | 15524 | 0.7966 | 0.9926 | 0.7754 | 0.9651 |
| H3_2 | 15335 | 0.3475 | 0.7991 | 0.7825 | 0.9651 |
| H3_2 | 15267 | 0.2563 | 0.7752 | 0.8006 | 0.9651 |
| H2A | 13865 | 0.2811 | 0.7763 | 0.8090 | 0.9651 |
| H3_1 | 15416 | 0.9021 | 0.9996 | 0.8097 | 0.9651 |
| H2B | 13977 | 0.3604 | 0.8162 | 0.8125 | 0.9651 |
| H2A | 14116 | 0.5989 | 0.9531 | 0.8132 | 0.9651 |
| H3_2 | 15503 | 0.4959 | 0.9233 | 0.8179 | 0.9651 |
| H4 | 10974 | 0.4482 | 0.9082 | 0.8229 | 0.9651 |
| H4 | 11363 | 0.8541 | 0.9996 | 0.8230 | 0.9651 |
| H3_1 | 15410 | 0.6447 | 0.971 | 0.8283 | 0.9651 |
| H4 | 11535 | 0.5751 | 0.9489 | 0.8332 | 0.9651 |
| H3_2 | 15510 | 0.8069 | 0.9926 | 0.8335 | 0.9651 |
| H4 | 11531 | 0.8680 | 0.9996 | 0.8344 | 0.9651 |
| H3_1 | 15466 | 0.8945 | 0.9996 | 0.8417 | 0.9651 |
| H3_2 | 15421 | 0.9399 | 0.9996 | 0.8441 | 0.9651 |
| H2A | 13831 | 0.8259 | 0.9937 | 0.8445 | 0.9651 |
| H4 | 11076 | 0.7164 | 0.971 | 0.8538 | 0.9651 |
| H2A | 13875 | 0.6141 | 0.9531 | 0.8587 | 0.9651 |
| H4 | 11333 | 0.3917 | 0.8456 | 0.8642 | 0.9651 |
| H4 | 11265 | 0.8121 | 0.9926 | 0.8642 | 0.9651 |
| H3_1 | 15476 | 0.7416 | 0.9734 | 0.8702 | 0.9651 |
| H3_1 | 15501 | 0.5274 | 0.9276 | 0.8786 | 0.9651 |
| H4 | 10990 | 0.8254 | 0.9937 | 0.8843 | 0.9651 |
| H4 | 11124 | 0.7981 | 0.9926 | 0.8848 | 0.9651 |
| H4 | 11280 | 0.6061 | 0.9531 | 0.8849 | 0.9651 |
| H4 | 11306 | 0.9893 | 1 | 0.8849 | 0.9651 |
| H2A | 14142 | 0.8121 | 0.9926 | 0.8874 | 0.9651 |
| H2B | 14079 | 0.6504 | 0.971 | 0.8932 | 0.9651 |
| H4 | 11068 | 0.9356 | 0.9996 | 0.8952 | 0.9651 |
| H2B | 14021 | 0.5105 | 0.9233 | 0.8953 | 0.9651 |
| H4 | 11405 | 0.5425 | 0.9283 | 0.8985 | 0.9651 |
| H3_2 | 15453 | 0.6942 | 0.971 | 0.9033 | 0.9651 |
| H3_1 | 15505 | 0.8205 | 0.9937 | 0.9066 | 0.9651 |
| H3_2 | 15464 | 0.5392 | 0.9283 | 0.9171 | 0.9706 |
| H4 | 11034 | 0.6565 | 0.971 | 0.9370 | 0.9706 |
| H2A | 13996 | 0.7027 | 0.971 | 0.9396 | 0.9706 |
| H3_2 | 15276 | 1.0000 | 1 | 0.9404 | 0.9706 |
| H2B | 14055 | 0.9563 | 0.9996 | 0.9423 | 0.9706 |
| H3_2 | 15289 | 0.8766 | 0.9996 | 0.9501 | 0.9706 |
| H3_2 | 15378 | 1.0000 | 1 | 0.9505 | 0.9706 |
| H2A | 14070 | 0.7629 | 0.9901 | 0.9516 | 0.9706 |
| H2B | 14008 | 0.7901 | 0.9926 | 0.9578 | 0.9706 |
| H2B | 13911 | 0.7314 | 0.971 | 0.9579 | 0.9706 |
| H2B | 14067 | 0.7699 | 0.9926 | 0.9580 | 0.9706 |
| H3_1 | 15396 | 0.3103 | 0.7964 | 0.9654 | 0.9738 |
| H3_1 | 15480 | 0.8530 | 0.9996 | 0.9792 | 0.9835 |
| H4 | 11419 | 0.8401 | 0.9996 | 0.9895 | 0.9895 |

**Table 9- Histone isoforms-Summary results for treatment**

| Variant | Fragment | HR | LCL | UCL | Raw P | **FDR** |
| --- | --- | --- | --- | --- | --- | --- |
| H2A | 14063 | 5.03 | 1.87 | 13.52 | 0.0004 | 0.0924 |
| H4 | 11014 | 2.66 | 1.26 | 5.62 | 0.0078 | 0.5422 |
| H3_2 | 15371 | 2.70 | 1.20 | 6.09 | 0.0124 | 0.5422 |
| H4 | 11005 | 2.37 | 1.16 | 4.86 | 0.0150 | 0.5422 |
| H4 | 11076 | 2.54 | 1.17 | 5.50 | 0.0153 | 0.5422 |
| H3_1 | 15289 | 0.37 | 0.16 | 0.87 | 0.0186 | 0.5422 |
| H3_1 | 15325 | 0.40 | 0.18 | 0.89 | 0.0201 | 0.5422 |
| H4 | 11002 | 0.44 | 0.21 | 0.90 | 0.0203 | 0.5422 |
| H3_1 | 15332 | 2.50 | 1.11 | 5.61 | 0.0222 | 0.5422 |
| H4 | 11433 | 0.46 | 0.22 | 0.93 | 0.0271 | 0.5422 |
| H3_2 | 15499 | 2.42 | 1.08 | 5.44 | 0.0279 | 0.5422 |
| H2A | 14197 | 0.45 | 0.21 | 0.95 | 0.0316 | 0.5422 |
| H3_2 | 15468 | 2.41 | 1.05 | 5.55 | 0.0333 | 0.5422 |
| H2A | 13916 | 0.46 | 0.22 | 0.96 | 0.0342 | 0.5422 |
| H2A | 14132 | 0.47 | 0.23 | 0.97 | 0.0362 | 0.5422 |
| H3_2 | 15449 | 2.36 | 1.02 | 5.43 | 0.0395 | 0.5422 |
| H3_2 | 15489 | 2.30 | 1.02 | 5.20 | 0.0399 | 0.5422 |
| H2A | 13911 | 0.49 | 0.24 | 1.02 | 0.0501 | 0.643 |
| H3_2 | 15442 | 2.11 | 0.96 | 4.68 | 0.0595 | 0.7234 |
| H3_1 | 15339 | 0.48 | 0.22 | 1.06 | 0.0634 | 0.7323 |
| H3_2 | 15400 | 2.04 | 0.92 | 4.53 | 0.0721 | 0.7707 |
| H3_1 | 15416 | 0.47 | 0.20 | 1.10 | 0.0734 | 0.7707 |
| H2A | 14046 | 1.93 | 0.92 | 4.04 | 0.0779 | 0.7824 |
| H3_2 | 15428 | 1.97 | 0.89 | 4.37 | 0.0883 | 0.8499 |
| H3_2 | 15386 | 1.95 | 0.88 | 4.30 | 0.0929 | 0.8582 |
| H4 | 11484 | 0.55 | 0.27 | 1.14 | 0.1011 | 0.8582 |
| H4 | 11497 | 0.55 | 0.26 | 1.15 | 0.1066 | 0.8582 |
| H2A | 14096 | 0.56 | 0.27 | 1.15 | 0.1073 | 0.8582 |
| H4 | 11384 | 0.57 | 0.28 | 1.15 | 0.1109 | 0.8582 |
| H3_2 | 15365 | 0.52 | 0.22 | 1.19 | 0.1133 | 0.8582 |
| H2A | 13958 | 1.78 | 0.86 | 3.70 | 0.1176 | 0.8582 |
| H2B | 13881 | 1.71 | 0.84 | 3.48 | 0.1328 | 0.8582 |
| H3_1 | 15303 | 0.52 | 0.22 | 1.24 | 0.1344 | 0.8582 |
| H2B | 13971 | 1.69 | 0.84 | 3.41 | 0.1380 | 0.8582 |
| H4 | 11535 | 1.72 | 0.83 | 3.55 | 0.1387 | 0.8582 |
| H2B | 14055 | 1.69 | 0.83 | 3.42 | 0.1413 | 0.8582 |
| H4 | 11461 | 1.69 | 0.83 | 3.46 | 0.1444 | 0.8582 |
| H2A | 14025 | 1.70 | 0.83 | 3.50 | 0.1447 | 0.8582 |
| H2A | 14018 | 1.71 | 0.81 | 3.62 | 0.1537 | 0.8582 |
| H4 | 11043 | 1.64 | 0.80 | 3.39 | 0.1734 | 0.8582 |
| H4 | 11048 | 0.62 | 0.30 | 1.25 | 0.1762 | 0.8582 |
| H4 | 11034 | 1.63 | 0.80 | 3.34 | 0.1776 | 0.8582 |
| H2B | 14032 | 0.55 | 0.22 | 1.34 | 0.1826 | 0.8582 |
| H2A | 13865 | 1.65 | 0.78 | 3.45 | 0.1835 | 0.8582 |
| H3_2 | 15524 | 0.59 | 0.27 | 1.30 | 0.1868 | 0.8582 |
| H3_1 | 15367 | 0.59 | 0.27 | 1.31 | 0.1892 | 0.8582 |
| H2B | 14002 | 1.61 | 0.78 | 3.33 | 0.1920 | 0.8582 |
| H3_1 | 15267 | 0.60 | 0.27 | 1.31 | 0.1935 | 0.8582 |
| H3_2 | 15344 | 1.67 | 0.75 | 3.70 | 0.2011 | 0.8582 |
| H2A | 13943 | 0.54 | 0.21 | 1.43 | 0.2087 | 0.8582 |
| H4 | 11292 | 0.65 | 0.32 | 1.29 | 0.2138 | 0.8582 |
| H2B | 14008 | 0.64 | 0.31 | 1.31 | 0.2155 | 0.8582 |
| H3_1 | 15424 | 0.62 | 0.28 | 1.34 | 0.2156 | 0.8582 |
| H3_1 | 15527 | 1.68 | 0.73 | 3.87 | 0.2171 | 0.8582 |
| H2B | 14092 | 0.64 | 0.32 | 1.31 | 0.2174 | 0.8582 |
| H2A | 14184 | 1.56 | 0.77 | 3.17 | 0.2186 | 0.8582 |
| H3_2 | 15506 | 1.65 | 0.74 | 3.70 | 0.2196 | 0.8582 |
| H4 | 11265 | 1.54 | 0.76 | 3.12 | 0.2272 | 0.8582 |
| H2A | 13890 | 0.65 | 0.32 | 1.33 | 0.2372 | 0.8582 |
| H3_1 | 15404 | 0.63 | 0.29 | 1.37 | 0.2372 | 0.8582 |
| H2B | 13747 | 1.53 | 0.75 | 3.10 | 0.2377 | 0.8582 |
| H3_2 | 15414 | 1.61 | 0.73 | 3.55 | 0.2378 | 0.8582 |
| H4 | 10965 | 0.65 | 0.32 | 1.34 | 0.2395 | 0.8582 |
| H3_2 | 15485 | 1.58 | 0.73 | 3.44 | 0.2417 | 0.8582 |
| H3_2 | 15503 | 0.60 | 0.25 | 1.44 | 0.2496 | 0.8582 |
| H3_2 | 15276 | 1.59 | 0.71 | 3.54 | 0.2545 | 0.8582 |
| H3_2 | 15289 | 1.56 | 0.72 | 3.41 | 0.2580 | 0.8582 |
| H3_1 | 15515 | 1.57 | 0.71 | 3.50 | 0.2623 | 0.8582 |
| H3_2 | 15279 | 1.84 | 0.62 | 5.50 | 0.2658 | 0.8582 |
| H4 | 11405 | 0.67 | 0.33 | 1.36 | 0.2672 | 0.8582 |
| H2B | 13805 | 1.51 | 0.73 | 3.13 | 0.2679 | 0.8582 |
| H3_1 | 15457 | 1.58 | 0.69 | 3.59 | 0.2719 | 0.8582 |
| H3_1 | 15476 | 0.65 | 0.29 | 1.43 | 0.2755 | 0.8582 |
| H3_2 | 15298 | 1.52 | 0.70 | 3.31 | 0.2847 | 0.8582 |
| H3_2 | 15314 | 1.53 | 0.69 | 3.40 | 0.2899 | 0.8582 |
| H3_1 | 15510 | 0.66 | 0.30 | 1.45 | 0.2922 | 0.8582 |
| H2B | 14021 | 0.68 | 0.33 | 1.41 | 0.2979 | 0.8582 |
| H3_1 | 15489 | 0.66 | 0.30 | 1.45 | 0.2998 | 0.8582 |
| H3_1 | 15485 | 1.51 | 0.69 | 3.28 | 0.3011 | 0.8582 |
| H4 | 11363 | 1.43 | 0.71 | 2.89 | 0.3167 | 0.8582 |
| H2A | 13899 | 1.42 | 0.70 | 2.89 | 0.3239 | 0.8582 |
| H4 | 11438 | 0.71 | 0.35 | 1.41 | 0.3241 | 0.8582 |
| H2B | 13759 | 0.71 | 0.35 | 1.42 | 0.3253 | 0.8582 |
| H2B | 13934 | 1.41 | 0.70 | 2.84 | 0.3309 | 0.8582 |
| H3_2 | 15407 | 1.48 | 0.67 | 3.26 | 0.3319 | 0.8582 |
| H3_1 | 15522 | 0.68 | 0.31 | 1.51 | 0.3403 | 0.8582 |
| H2B | 13987 | 1.41 | 0.69 | 2.87 | 0.3405 | 0.8582 |
| H2B | 14083 | 0.71 | 0.34 | 1.45 | 0.3421 | 0.8582 |
| H2B | 14067 | 1.40 | 0.69 | 2.85 | 0.3448 | 0.8582 |
| H2A | 13932 | 1.42 | 0.68 | 2.96 | 0.3493 | 0.8582 |
| H3_2 | 15392 | 1.52 | 0.62 | 3.73 | 0.3541 | 0.8582 |
| H2A | 14157 | 0.72 | 0.36 | 1.45 | 0.3546 | 0.8582 |
| H2B | 14095 | 0.72 | 0.35 | 1.46 | 0.3562 | 0.8582 |
| H3_2 | 15306 | 1.45 | 0.65 | 3.20 | 0.3604 | 0.8582 |
| H3_2 | 15519 | 1.44 | 0.66 | 3.16 | 0.3606 | 0.8582 |
| H2A | 13831 | 0.72 | 0.36 | 1.46 | 0.3608 | 0.8582 |
| H3_1 | 15296 | 0.69 | 0.31 | 1.53 | 0.3624 | 0.8582 |
| H4 | 11250 | 1.37 | 0.69 | 2.73 | 0.3676 | 0.8582 |
| H3_2 | 15358 | 1.43 | 0.65 | 3.17 | 0.3693 | 0.8582 |
| H3_2 | 15528 | 1.43 | 0.65 | 3.17 | 0.3715 | 0.8582 |
| H4 | 11068 | 1.37 | 0.68 | 2.77 | 0.3840 | 0.8634 |
| H3_1 | 15347 | 1.50 | 0.60 | 3.76 | 0.3877 | 0.8634 |
| H4 | 11039 | 0.74 | 0.37 | 1.47 | 0.3909 | 0.8634 |
| H2B | 13831 | 0.74 | 0.36 | 1.50 | 0.4006 | 0.8634 |
| H4 | 11306 | 0.75 | 0.37 | 1.51 | 0.4189 | 0.8634 |
| H2A | 13858 | 0.75 | 0.37 | 1.52 | 0.4278 | 0.8634 |
| H3_2 | 15534 | 1.37 | 0.62 | 3.03 | 0.4298 | 0.8634 |
| H2A | 13817 | 0.75 | 0.36 | 1.55 | 0.4299 | 0.8634 |
| H3_1 | 15543 | 0.73 | 0.33 | 1.62 | 0.4368 | 0.8634 |
| H3_1 | 15533 | 0.73 | 0.33 | 1.62 | 0.4397 | 0.8634 |
| H2B | 14048 | 1.32 | 0.65 | 2.69 | 0.4403 | 0.8634 |
| H4 | 11028 | 1.31 | 0.65 | 2.65 | 0.4487 | 0.8634 |
| H2A | 14083 | 0.75 | 0.36 | 1.59 | 0.4544 | 0.8634 |
| H3_1 | 15505 | 0.74 | 0.34 | 1.63 | 0.4548 | 0.8634 |
| H2B | 13963 | 0.77 | 0.38 | 1.54 | 0.4556 | 0.8634 |
| H3_2 | 15267 | 0.74 | 0.33 | 1.64 | 0.4580 | 0.8634 |
| H3_2 | 15378 | 1.37 | 0.60 | 3.12 | 0.4593 | 0.8634 |
| H3_1 | 15260 | 1.34 | 0.61 | 2.92 | 0.4666 | 0.8634 |
| H3_2 | 15461 | 1.50 | 0.50 | 4.51 | 0.4711 | 0.8634 |
| H3_2 | 15335 | 1.33 | 0.61 | 2.88 | 0.4730 | 0.8634 |
| H2A | 14057 | 0.78 | 0.39 | 1.56 | 0.4743 | 0.8634 |
| H2A | 14142 | 0.77 | 0.37 | 1.58 | 0.4749 | 0.8634 |
| H2A | 14112 | 0.77 | 0.38 | 1.57 | 0.4750 | 0.8634 |
| H4 | 11419 | 1.29 | 0.64 | 2.61 | 0.4785 | 0.8634 |
| H3_1 | 15547 | 0.75 | 0.34 | 1.68 | 0.4826 | 0.8634 |
| H2B | 13921 | 1.28 | 0.64 | 2.58 | 0.4853 | 0.8634 |
| H2B | 13957 | 0.78 | 0.39 | 1.58 | 0.4883 | 0.8634 |
| H3_2 | 15514 | 1.31 | 0.60 | 2.84 | 0.4940 | 0.8634 |
| H3_1 | 15431 | 1.41 | 0.52 | 3.82 | 0.4942 | 0.8634 |
| H2B | 13977 | 0.78 | 0.38 | 1.59 | 0.4965 | 0.8634 |
| H3_2 | 15294 | 0.74 | 0.30 | 1.79 | 0.4975 | 0.8634 |
| H4 | 11373 | 1.27 | 0.63 | 2.55 | 0.4994 | 0.8634 |
| H4 | 11546 | 1.28 | 0.63 | 2.60 | 0.5025 | 0.8634 |
| H3_2 | 15548 | 1.31 | 0.60 | 2.86 | 0.5030 | 0.8634 |
| H4 | 11516 | 0.79 | 0.39 | 1.58 | 0.5046 | 0.8634 |
| H4 | 11056 | 1.26 | 0.63 | 2.49 | 0.5153 | 0.8753 |
| H3_1 | 15444 | 1.30 | 0.59 | 2.87 | 0.5228 | 0.8798 |
| H2A | 14147 | 0.80 | 0.39 | 1.61 | 0.5256 | 0.8798 |
| H3_2 | 15493 | 1.27 | 0.58 | 2.77 | 0.5483 | 0.8986 |
| H3_1 | 15390 | 0.78 | 0.34 | 1.80 | 0.5553 | 0.8986 |
| H2A | 14116 | 1.23 | 0.61 | 2.47 | 0.5614 | 0.8986 |
| H4 | 11531 | 0.82 | 0.41 | 1.62 | 0.5658 | 0.8986 |
| H3_1 | 15353 | 0.79 | 0.36 | 1.75 | 0.5669 | 0.8986 |
| H4 | 11322 | 0.82 | 0.41 | 1.63 | 0.5679 | 0.8986 |
| H2B | 13819 | 1.23 | 0.60 | 2.50 | 0.5689 | 0.8986 |
| H2B | 13928 | 1.22 | 0.61 | 2.46 | 0.5696 | 0.8986 |
| H3_1 | 15537 | 1.25 | 0.57 | 2.73 | 0.5739 | 0.8986 |
| H2B | 13790 | 0.82 | 0.41 | 1.65 | 0.5774 | 0.8986 |
| H4 | 11488 | 1.22 | 0.61 | 2.45 | 0.5796 | 0.8986 |
| H3_1 | 15501 | 0.80 | 0.37 | 1.76 | 0.5837 | 0.8989 |
| H3_1 | 15498 | 0.80 | 0.35 | 1.82 | 0.5942 | 0.909 |
| H4 | 10990 | 0.83 | 0.42 | 1.66 | 0.6020 | 0.9114 |
| H2B | 13940 | 0.83 | 0.41 | 1.68 | 0.6066 | 0.9114 |
| H3_2 | 15472 | 0.81 | 0.36 | 1.82 | 0.6076 | 0.9114 |
| H4 | 11333 | 0.84 | 0.42 | 1.68 | 0.6138 | 0.9148 |
| H4 | 10974 | 0.84 | 0.42 | 1.68 | 0.6230 | 0.9225 |
| H4 | 11542 | 0.84 | 0.42 | 1.71 | 0.6370 | 0.9298 |
| H3_1 | 15281 | 0.83 | 0.39 | 1.80 | 0.6396 | 0.9298 |
| H2A | 13906 | 1.19 | 0.58 | 2.42 | 0.6400 | 0.9298 |
| H2B | 13775 | 0.85 | 0.42 | 1.73 | 0.6526 | 0.9331 |
| H3_1 | 15434 | 0.81 | 0.32 | 2.04 | 0.6528 | 0.9331 |
| H3_2 | 15303 | 0.81 | 0.32 | 2.04 | 0.6544 | 0.9331 |
| H3_1 | 15410 | 0.84 | 0.38 | 1.86 | 0.6698 | 0.942 |
| H3_1 | 15310 | 0.84 | 0.38 | 1.86 | 0.6723 | 0.942 |
| H3_1 | 15472 | 0.85 | 0.39 | 1.86 | 0.6875 | 0.942 |
| H4 | 11473 | 0.87 | 0.43 | 1.74 | 0.6896 | 0.942 |
| H3_1 | 15480 | 0.86 | 0.40 | 1.85 | 0.6916 | 0.942 |
| H2A | 14168 | 1.15 | 0.57 | 2.33 | 0.6945 | 0.942 |
| H3_1 | 15438 | 1.16 | 0.54 | 2.52 | 0.7018 | 0.942 |
| H2B | 13902 | 1.15 | 0.56 | 2.36 | 0.7083 | 0.942 |
| H2B | 13873 | 1.14 | 0.57 | 2.27 | 0.7204 | 0.942 |
| H3_2 | 15478 | 1.15 | 0.53 | 2.50 | 0.7224 | 0.942 |
| H4 | 10921 | 0.88 | 0.44 | 1.79 | 0.7302 | 0.942 |
| H2A | 14103 | 0.89 | 0.43 | 1.82 | 0.7401 | 0.942 |
| H4 | 11124 | 1.12 | 0.57 | 2.23 | 0.7420 | 0.942 |
| H3_2 | 15321 | 1.14 | 0.52 | 2.51 | 0.7424 | 0.942 |
| H4 | 11280 | 1.12 | 0.56 | 2.26 | 0.7437 | 0.942 |
| H2B | 13877 | 0.89 | 0.43 | 1.84 | 0.7449 | 0.942 |
| H4 | 11378 | 0.89 | 0.45 | 1.77 | 0.7458 | 0.942 |
| H3_1 | 15466 | 1.14 | 0.51 | 2.52 | 0.7517 | 0.942 |
| H3_2 | 15421 | 1.14 | 0.47 | 2.80 | 0.7710 | 0.942 |
| H2A | 13875 | 1.11 | 0.55 | 2.23 | 0.7726 | 0.942 |
| H3_2 | 15283 | 1.12 | 0.52 | 2.42 | 0.7786 | 0.942 |
| H3_2 | 15272 | 1.12 | 0.51 | 2.46 | 0.7797 | 0.942 |
| H3_1 | 15494 | 1.12 | 0.51 | 2.45 | 0.7833 | 0.942 |
| H3_2 | 15538 | 0.90 | 0.41 | 1.96 | 0.7839 | 0.942 |
| H2A | 14173 | 0.91 | 0.45 | 1.83 | 0.7841 | 0.942 |
| H3_2 | 15543 | 1.11 | 0.51 | 2.42 | 0.7865 | 0.942 |
| H2B | 13844 | 0.91 | 0.45 | 1.85 | 0.7882 | 0.942 |
| H3_2 | 15436 | 0.88 | 0.35 | 2.25 | 0.7950 | 0.942 |
| H4 | 11389 | 0.91 | 0.46 | 1.81 | 0.7961 | 0.942 |
| H2B | 13898 | 0.91 | 0.45 | 1.86 | 0.7966 | 0.942 |
| H4 | 10946 | 0.92 | 0.46 | 1.82 | 0.8006 | 0.942 |
| H3_1 | 15448 | 0.90 | 0.41 | 2.00 | 0.8036 | 0.942 |
| H2B | 13944 | 1.09 | 0.54 | 2.20 | 0.8046 | 0.942 |
| H3_1 | 15382 | 0.91 | 0.41 | 2.00 | 0.8082 | 0.942 |
| H2A | 13996 | 1.09 | 0.53 | 2.24 | 0.8109 | 0.942 |
| H2A | 14070 | 1.09 | 0.54 | 2.21 | 0.8161 | 0.942 |
| H2B | 13839 | 0.92 | 0.46 | 1.85 | 0.8161 | 0.942 |
| H3_1 | 15461 | 1.09 | 0.51 | 2.37 | 0.8193 | 0.942 |
| H3_2 | 15510 | 1.09 | 0.51 | 2.37 | 0.8198 | 0.942 |
| H3_2 | 15261 | 1.10 | 0.50 | 2.42 | 0.8237 | 0.942 |
| H2A | 13940 | 1.08 | 0.53 | 2.17 | 0.8361 | 0.9514 |
| H3_1 | 15317 | 0.92 | 0.42 | 2.04 | 0.8430 | 0.9546 |
| H3_1 | 15396 | 1.08 | 0.49 | 2.38 | 0.8519 | 0.9564 |
| H4 | 11503 | 1.07 | 0.53 | 2.16 | 0.8558 | 0.9564 |
| H2A | 14004 | 0.94 | 0.47 | 1.89 | 0.8570 | 0.9564 |
| H3_1 | 15376 | 1.08 | 0.46 | 2.50 | 0.8647 | 0.957 |
| H4 | 11348 | 0.94 | 0.47 | 1.88 | 0.8659 | 0.957 |
| H2B | 13855 | 0.95 | 0.47 | 1.90 | 0.8733 | 0.9572 |
| H3_2 | 15329 | 1.07 | 0.48 | 2.37 | 0.8743 | 0.9572 |
| H3_2 | 15457 | 1.06 | 0.49 | 2.30 | 0.8887 | 0.9683 |
| H2A | 13987 | 0.96 | 0.47 | 1.94 | 0.9047 | 0.9801 |
| H3_2 | 15256 | 1.05 | 0.48 | 2.28 | 0.9086 | 0.9801 |
| H3_2 | 15453 | 0.96 | 0.44 | 2.07 | 0.9159 | 0.9801 |
| H4 | 11019 | 1.04 | 0.52 | 2.07 | 0.9189 | 0.9801 |
| H4 | 11511 | 1.04 | 0.51 | 2.12 | 0.9207 | 0.9801 |
| H3_1 | 15274 | 1.04 | 0.43 | 2.47 | 0.9371 | 0.993 |
| H2A | 13847 | 1.03 | 0.51 | 2.06 | 0.9420 | 0.9934 |
| H2B | 14079 | 0.98 | 0.49 | 1.96 | 0.9507 | 0.9934 |
| H2A | 14033 | 1.02 | 0.50 | 2.08 | 0.9580 | 0.9934 |
| H4 | 11526 | 0.98 | 0.50 | 1.95 | 0.9607 | 0.9934 |
| H2B | 13911 | 1.02 | 0.50 | 2.07 | 0.9621 | 0.9934 |
| H4 | 11519 | 0.99 | 0.48 | 2.01 | 0.9669 | 0.9934 |
| H3_2 | 15264 | 1.02 | 0.45 | 2.29 | 0.9676 | 0.9934 |
| H3_2 | 15351 | 0.99 | 0.46 | 2.15 | 0.9788 | 0.9958 |
| H2B | 13888 | 1.01 | 0.50 | 2.02 | 0.9801 | 0.9958 |
| H3_2 | 15464 | 1.01 | 0.38 | 2.71 | 0.9841 | 0.9958 |
| H3_1 | 15452 | 1.00 | 0.46 | 2.18 | 0.9942 | 0.9958 |
| H2B | 13952 | 1.00 | 0.50 | 2.01 | 0.9950 | 0.9958 |
| H4 | 11446 | 1.00 | 0.50 | 2.00 | 0.9958 | 0.9958 |
