# Supplementary figures and images for: Proteomic profiling identifies specific histone species associated with leukemic and cancer cells
Source: Clin Proteomics. 2015 Aug 27;12(1):22. doi: 10.1186/s12014-015-9095-4 (PMC4551702; doi:10.1186/s12014-015-9095-4)

## Slide 1
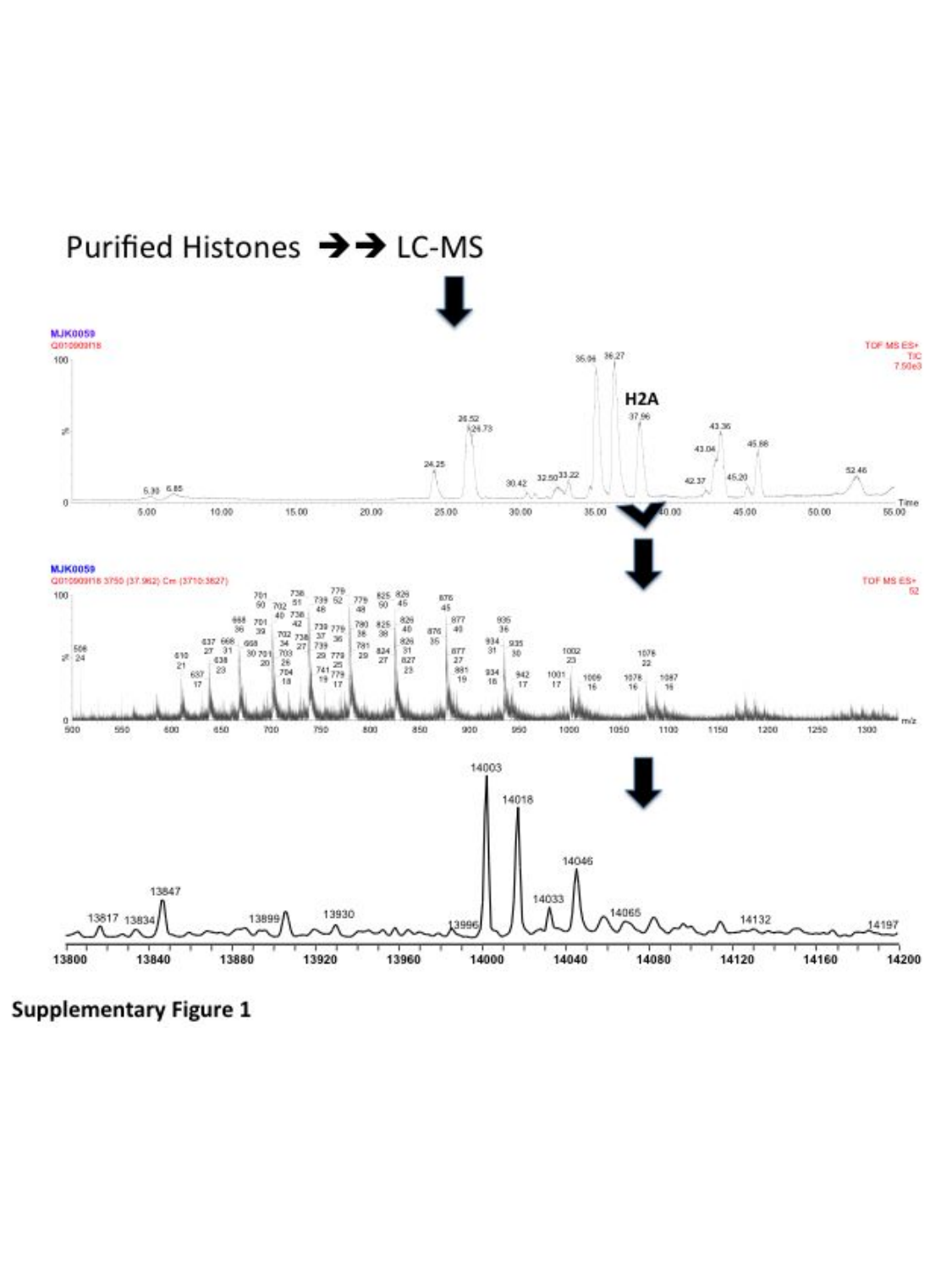

## Slide 2
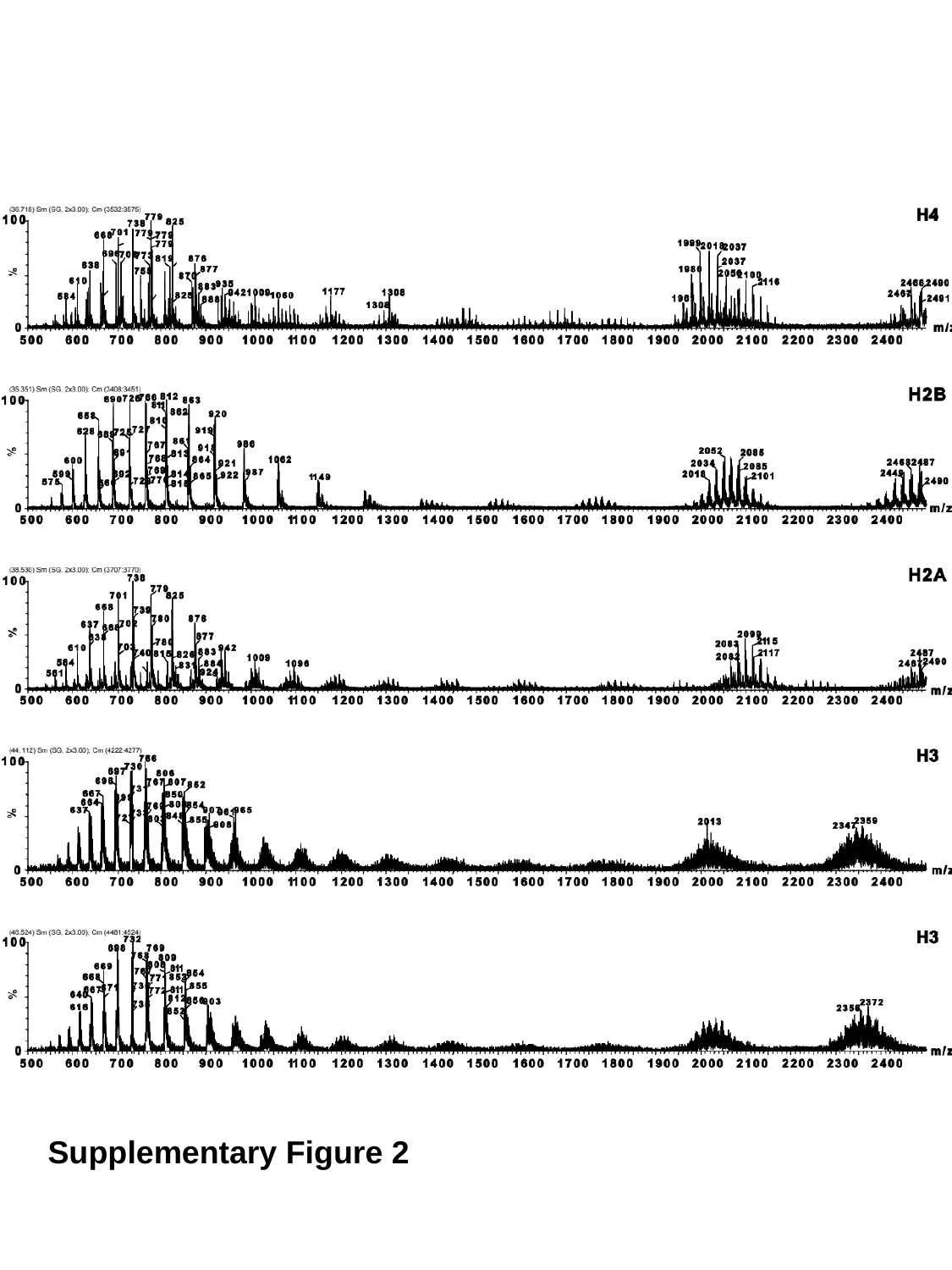

Supplementary Figure 2

## Slide 3
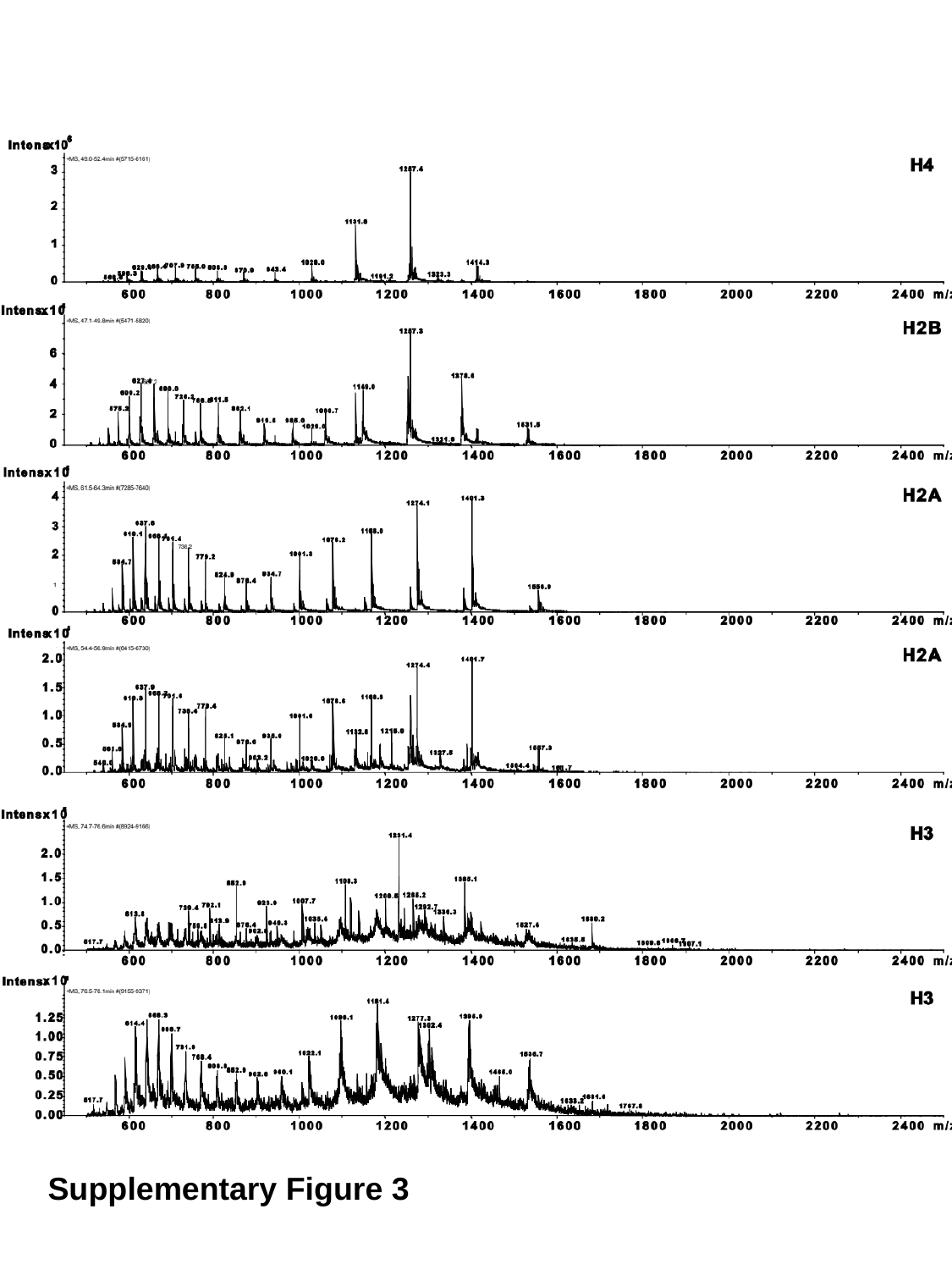

Supplementary Figure 3

Supplement: Additional file 2: — Figure S1. A schematic diagram of the method used to quantitate histone proteomics in tumor samples. Figures S2, S3. Examples of raw spectra. [file 12014_2015_9095_MOESM2_ESM.pptx]
